# IgM anti-ACE2 autoantibodies in severe COVID-19 activate complement and perturb vascular endothelial function

**DOI:** 10.1172/jci.insight.158362

**Published:** 2022-05-09

**Authors:** Livia Casciola-Rosen, David R. Thiemann, Felipe Andrade, Maria I. Trejo-Zambrano, Elissa K. Leonard, Jamie B. Spangler, Nicole E. Skinner, Justin Bailey, Srinivasan Yegnasubramanian, Rulin Wang, Ajay M. Vaghasia, Anuj Gupta, Andrea L. Cox, Stuart C. Ray, Raleigh M. Linville, Zhaobin Guo, Peter C. Searson, Carolyn E. Machamer, Stephen Desiderio, Lauren M. Sauer, Oliver Laeyendecker, Brian T. Garibaldi, Li Gao, Mahendra Damarla, Paul M. Hassoun, Jody E. Hooper, Christopher A. Mecoli, Lisa Christopher-Stine, Laura Gutierrez-Alamillo, Qingyuan Yang, David Hines, William A. Clarke, Richard E. Rothman, Andrew Pekosz, Katherine Z.J. Fenstermacher, Zitong Wang, Scott L. Zeger, Antony Rosen

**Affiliations:** 1 Department of Medicine, Division of Rheumatology;; 2 Department of Medicine, Division of Cardiology; and; 3 Department of Biomedical Engineering, Johns Hopkins University School of Medicine, Baltimore, Maryland, USA.; 4 Department of Chemical and Biomolecular Engineering, Whiting School of Engineering, Johns Hopkins University, Baltimore, Maryland, USA.; 5 Translational Tissue Engineering Center;; 6 Department of Medicine, Division of Infectious Diseases; and; 7 Department of Oncology, Johns Hopkins University School of Medicine, Baltimore, Maryland, USA.; 8 Institute for NanoBioTechnology and; 9 Department of Materials Science and Engineering, Whiting School of Engineering, Johns Hopkins University, Baltimore, Maryland, USA.; 10 Department of Cell Biology and; 11 Department of Molecular Biology and Genetics, Johns Hopkins University School of Medicine, Baltimore, Maryland, USA.; 12 Adult Emergency Department, Johns Hopkins Hospital, Baltimore, Maryland, USA.; 13 Johns Hopkins Biocontainment Unit, Johns Hopkins University School of Medicine, Baltimore, Maryland, USA.; 14 Division of Intramural Medicine, National Institute of Allergy and Infectious Diseases, NIH, Baltimore, Maryland, USA.; 15 Department of Medicine, Division of Pulmonary and Critical Care Medicine;; 16 Department of Medicine, Division of Allergy and Clinical Immunology; and; 17 Department of Pathology, Johns Hopkins University School of Medicine, Baltimore, Maryland, USA.; 18 Department of Environmental Health and Engineering,; 19 Department of Molecular Microbiology and Immunology, and; 20 Department of Biostatistics, Bloomberg School of Public Health, Johns Hopkins University, Baltimore, Maryland, USA.

**Keywords:** Autoimmunity, COVID-19, Rheumatology

## Abstract

**Background:**

Some clinical features of severe COVID-19 represent blood vessel damage induced by activation of host immune responses initiated by the coronavirus SARS-CoV-2. We hypothesized autoantibodies against angiotensin-converting enzyme 2 (ACE2), the SARS-CoV-2 receptor expressed on vascular endothelium, are generated during COVID-19 and are of mechanistic importance.

**Methods:**

In an opportunity sample of 118 COVID-19 inpatients, autoantibodies recognizing ACE2 were detected by ELISA. Binding properties of anti-ACE2 IgM were analyzed via biolayer interferometry. Effects of anti-ACE2 IgM on complement activation and endothelial function were demonstrated in a tissue-engineered pulmonary microvessel model.

**Results:**

Anti-ACE2 IgM (not IgG) autoantibodies were associated with severe COVID-19 and found in 18/66 (27.2%) patients with severe disease compared with 2/52 (3.8%) of patients with moderate disease (OR 9.38, 95% CI 2.38–42.0; *P* = 0.0009). Anti-ACE2 IgM autoantibodies were rare (2/50) in non-COVID-19 ventilated patients with acute respiratory distress syndrome. Unexpectedly, ACE2-reactive IgM autoantibodies in COVID-19 did not undergo class-switching to IgG and had apparent *K_D_* values of 5.6–21.7 nM, indicating they are T cell independent. Anti-ACE2 IgMs activated complement and initiated complement-binding and functional changes in endothelial cells in microvessels, suggesting they contribute to the angiocentric pathology of COVID-19.

**Conclusion:**

We identify anti-ACE2 IgM as a mechanism-based biomarker strongly associated with severe clinical outcomes in SARS-CoV-2 infection, which has therapeutic implications.

**FUNDING:**

Bill & Melinda Gates Foundation, Gates Philanthropy Partners, Donald B. and Dorothy L. Stabler Foundation, and Jerome L. Greene Foundation; NIH R01 AR073208, R01 AR069569, Institutional Research and Academic Career Development Award (5K12GM123914-03), National Heart, Lung, and Blood Institute R21HL145216, and Division of Intramural Research, National Institute of Allergy and Infectious Diseases; National Science Foundation Graduate Research Fellowship (DGE1746891)

## Graphical abstract

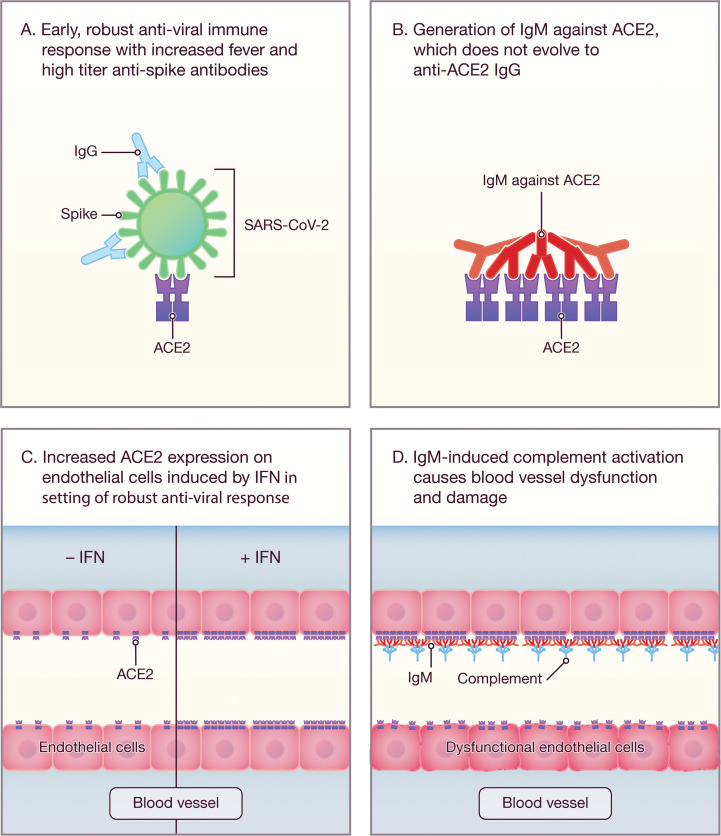

## Introduction

COVID-19 is caused by the novel coronavirus SARS-CoV-2 (1). This virus is a highly infectious pathogen and has had a massive global impact since its recognition in Wuhan, China, in late 2019, with more than 300 million confirmed infections, 5.5 million confirmed deaths, and disruption around the world. Although most infections in unvaccinated individuals appear to be self-limited, 15% to 20% of symptomatic individuals become hospitalized, and 5% to 10% require admission to intensive care units (2, 3). Two years into the pandemic, mortality rates of hospitalized patients with COVID-19 in the United States still exceeded 10%. The observation that a significant subgroup of COVID-19 patients experience the onset of severe manifestations in a second phase beginning 7 to 12 days after initial symptom development has focused attention on the mechanisms underlying severity in this second phase.

There is significant evidence for preexisting IgG autoantibodies against type I IFNs playing a key role in mediating disease severity in COVID-19 (4), likely by enabling enhanced viral pathogenicity. There is also evidence that some severe COVID-19 clinical features represent damage of blood vessels induced by activation of host immune and inflammatory responses, initiated by the virus, but not necessarily directed only at viral antigens (5–7). Multiple IgG autoantibodies recognizing additional immunomodulatory and extracellular proteins and phospholipids have been found, which are possible contributors to disease severity (8, 9). However, a role for IgM autoantibodies against vascular targets as mediators of vascular dysfunction in COVID-19 is unknown. Of particular note in this regard are several inflammatory syndromes where IgM that recognize various surface-exposed autoantigens, arising in the context of acute infections, induce significant pathology. Examples include postinfectious agglutinins that induce hemolysis (10), as well as “single-shot” autoimmune syndromes like Kawasaki syndrome, which target blood vessels (11). The finding that short-term, low-dose dexamethasone has a beneficial effect on mortality in a subgroup of patients with severe COVID-19 requiring oxygen suggests that uncontrolled inflammatory mechanisms might play an apical role in mediating disease severity in a subset of patients with this disease (12). Understanding the origin of such early inflammatory mechanisms may identify additional therapeutic strategies with impact in severe COVID-19.

## Results

### IgM autoantibodies recognizing angiotensin-converting enzyme 2 are associated with severe disease in COVID-19.

**Figure 1:**
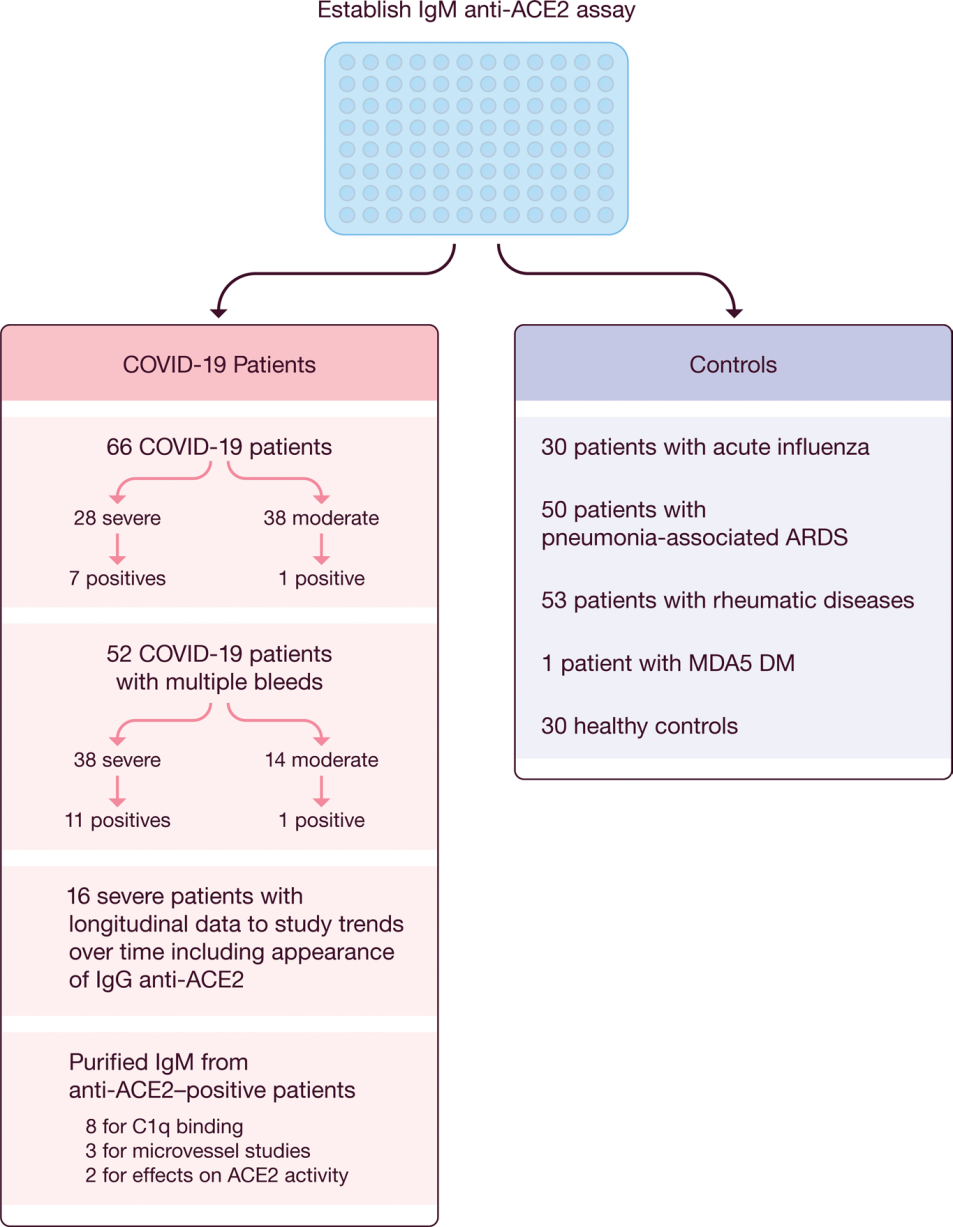
Flow diagram for identification of anti-ACE2 IgM antibodies. Anti-ACE2 IgM antibodies were assayed by ELISA in serum from 66 patients with COVID-19, 52 COVID-19 patients with multiple bleeds available, 133 disease controls, and 30 healthy controls. The functional consequences of these antibodies were investigated with 3 different assays using IgM purified from anti-ACE2 IgM–positive sera. MDA5 DM, melanoma differentiation-associated 5 dermatomyositis.

**Figure 2:**
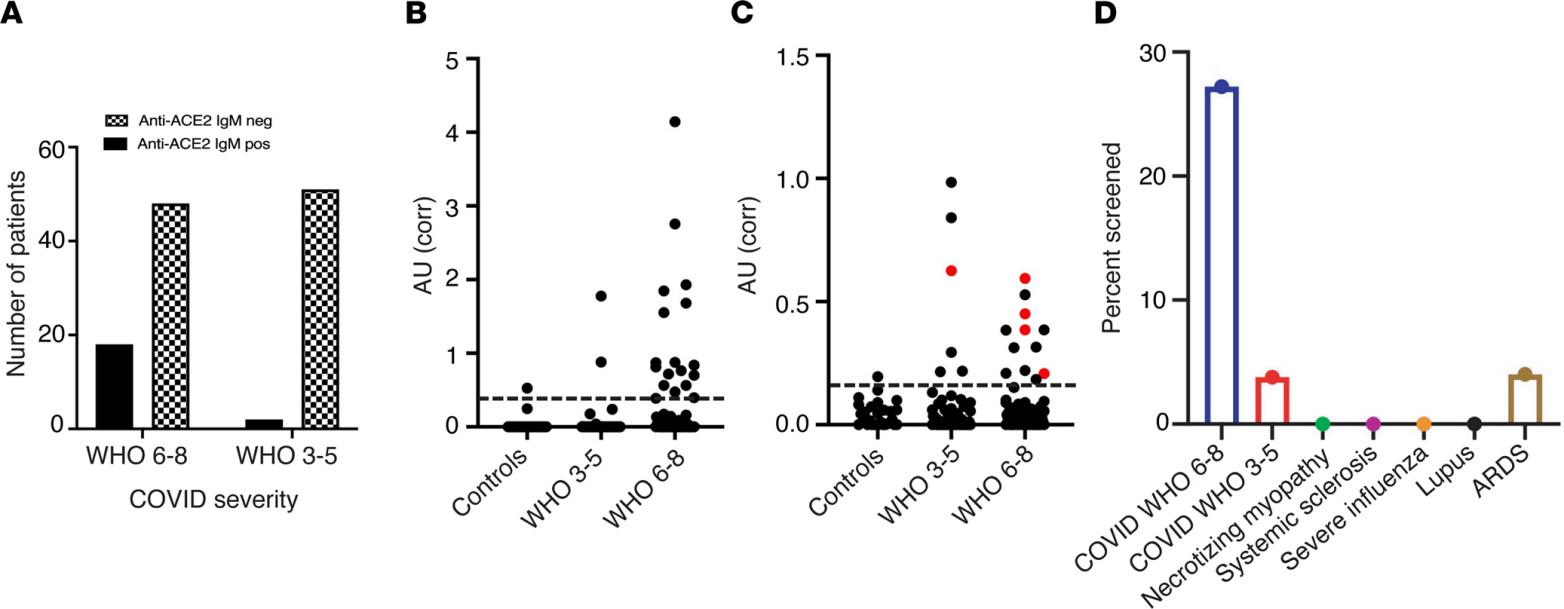
Anti-ACE2 IgM antibodies are found in patients with COVID-19. (**A**–**C**) Antibodies were assayed by ELISA in the combined COVID-19 cohort (*n* = 118 patients). (**A**) Number of patients with and without anti-ACE2 IgM antibodies shown grouped by disease severity: 27.2% of severe patients were anti-ACE2 positive compared with 3.8% with moderate COVID-19 (*P* = 0.0009; 2-tailed Fisher’s exact test). (**B** and **C**) Data from anti-ACE2 IgM (**B**) and IgG (**C**) ELISAs are presented as corrected OD 450 absorbance units. These data were obtained on all the patients with COVID-19 in **A**, as well as from 30 healthy controls. Red dots in the IgG panel denote IgG-positive samples that also have anti-ACE2 IgM antibodies. The horizontal line on each plot represents the cutoff for assigning a positive antibody status. (**D**) Anti-ACE2 IgM antibodies are detected in patients with COVID-19 but not in other infectious and autoimmune disease controls.

We hypothesized that angiotensin-converting enzyme 2 (ACE2), the host receptor for SARS-CoV-2 entry (1), could be a potential autoantigen in COVID-19. SARS-CoV-2 spike (S) protein binds to ACE2 with 5- to 20-fold higher affinities than the other coronaviruses that also bind to this host receptor (13). Furthermore, ACE2 expression is enhanced in the lung (epithelial and endothelial cells) and heart (endothelial cells; ref. 14), and hypomorphic ACE2 function has been implicated in adverse outcomes in models of acute respiratory distress syndrome (ARDS) (15). We therefore established assays to screen for IgM and IgG autoantibodies against ACE2 and applied these initially to a cohort of 66 hospitalized patients with COVID-19 who reached the 6 most severe WHO ordinal groups as their maximal severity (28 severe, 38 moderate) (see Figure 1 for a flow diagram summarizing the cohorts studied and findings and Methods for more details about the WHO groups). Eight patients were positive for anti-ACE2 IgM autoantibodies. Seven of these were in the mechanically ventilated (WHO 6/7) or dead groups (WHO 8) (7/28; 25%), while only a single patient was positive among the 38 patients who were never ventilated (1/38; 2.6%; OR 12.3, 95% CI 1.875–141.9; *P* = 0.0084 by Fisher’s exact test; Supplemental Figure 1A, left panel; supplemental material available online with this article; https://doi.org/10.1172/jci.insight.158362DS1). In order to increase sample size and define the stability and kinetics of these antibodies, we assembled additional patients in whom serum was available from multiple laboratory blood draws taken across their hospitalization. This added 52 patients with COVID-19 for serum analysis: 38 in WHO ordinal groups 6 to 8 (31 ventilated and 7 dead) and 14 patients in ordinal group 4 (demographics of the combined study population are presented in Supplemental Table 1). The frequencies of anti-ACE2 IgM in these patients were very similar to the initial group: 11/38 (28.9%) of the patients with severe COVID-19 were positive for anti-ACE2 IgM antibodies compared with 1/14 (7.1%) in the group with milder COVID-19 (Supplemental Figure 1A, right panel). The combined frequency of anti-ACE2 IgM in severe COVID-19 was 18/66 patients (27.2%) compared with 2/52 patients (3.8%) with moderate COVID-19 (OR 9.38, 95% CI 2.38–42.0; *P* = 0.0009 by Fisher’s exact test; Figure 2A). IgM levels were consistently well above the limits of detection (Figure 2B); all positives were confirmed and quantified by serial dilution (representative examples are shown in Supplemental Figure 1B).

In contrast to the findings with IgM, anti-ACE2 IgG autoantibodies were not enriched in severe disease, as found in 12/66 (18%) patients with severe COVID-19 (WHO 6–8) and 6/52 (11.5%) patients with moderate (WHO 3–5) disease (*P* = 0.44 by Fisher’s exact test). Only 4/18 (22%) severe patients with anti-ACE2 IgM antibodies were also IgG positive (Figure 2C). ACE2 is therefore a prominent autoantibody target in patients with COVID-19, with IgM autoantibodies more prevalent in severe as compared with moderate disease.

Controls with various infectious and autoimmune diseases were also tested for anti-ACE2 IgM (Figure 2D). Anti-ACE2 IgM autoantibodies were not observed in 30 patients with acute influenza infection (including 11 patients evaluated in the emergency department and discharged to outpatient care and 19 hospitalized patients requiring oxygen therapy or assisted ventilation; ref. 5). In addition, in a cohort of 50 ventilated patients with pneumonia-associated ARDS, only 2 (4%) had low-titer anti-ACE2 IgM. Similarly, 0 of 53 patients (0%) with autoimmune rheumatic diseases (25 patients with systemic lupus erythematosus [SLE], 13 with scleroderma, and 15 with autoimmune necrotizing myopathy) had IgM autoantibodies recognizing ACE2. Interestingly, ACE2 IgM autoantibodies were initially found in a banked serum sample from an index patient with a rare acute dermatopulmonary syndrome associated with autoantibodies against MDA5 (16), which appears to phenocopy several features of severe COVID-19 (see Methods; additional studies on similar patients are underway).

Because ACE2 is present in tetraspanin-enriched microdomains, where it clusters with other glycoproteins, such as CD9, CD81, and TMPRSS2, we also established assays to define whether anti-IgM antibodies against any of these proteins were present in COVID-19 sera. None of these molecules were recognized in anti-ACE2 IgM–positive or –negative patient sera (Supplemental Figure 2, A and B). Collectively, our data show a striking specificity for the association of anti-ACE2 IgM with severe COVID-19.

**Figure 3:**
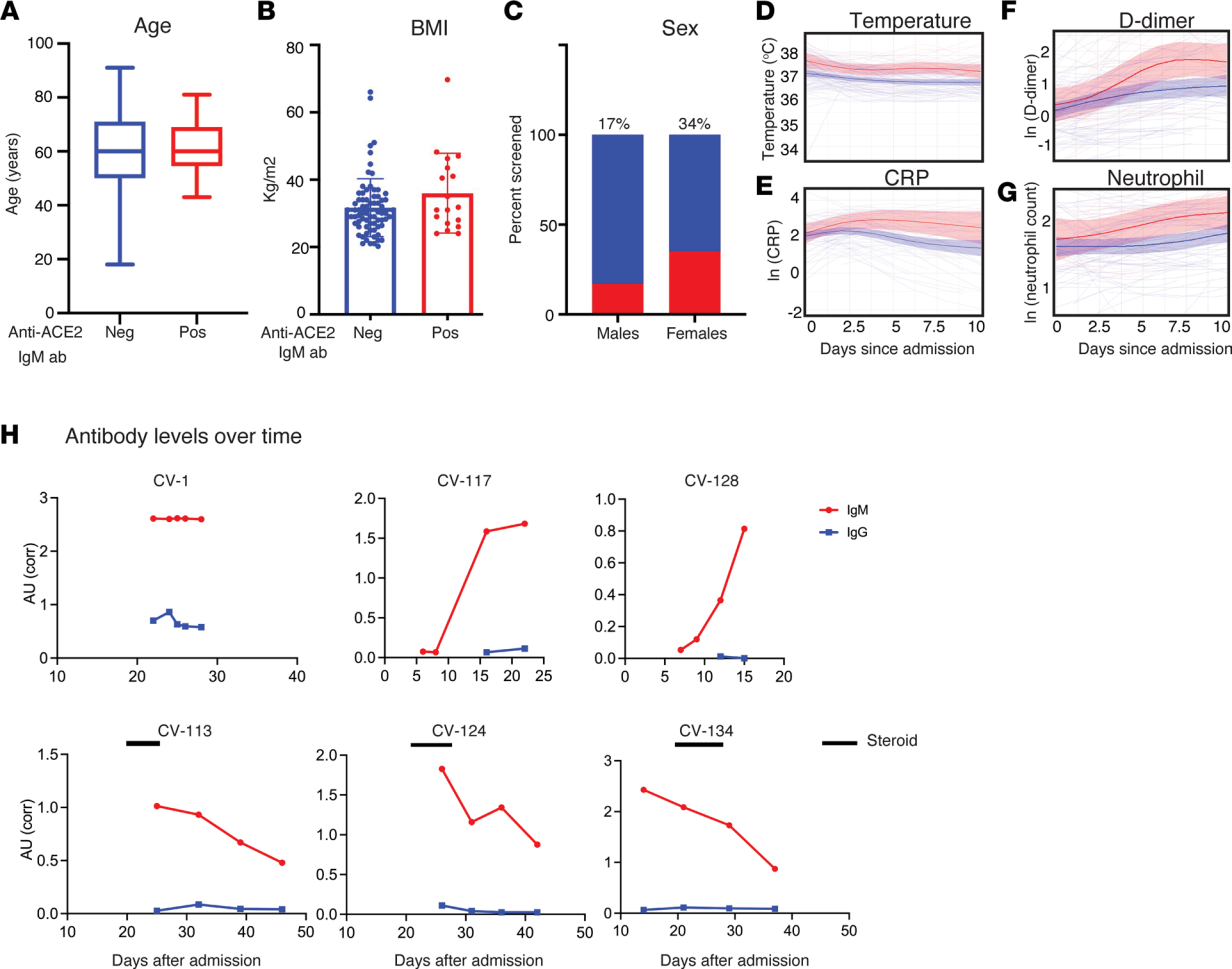
Clinical features of anti-ACE2 IgM–positive COVID-19 patients compared with those who do not have these antibodies. (**A**) Age, (**B**) BMI, (**C**) sex, and levels of (**D**) temperature, (**E**) CRP, (**F**) D-dimer, and (**G**) neutrophils were compared between the anti-ACE2 IgM–positive and –negative COVID-19 patient groups. Red and blue colors denote anti-ACE2 IgM antibody–positive and –negative status, respectively. Box plots show median, 25th and 75th percentiles, and whiskers min to max. (**D**–**G**) Anti-ACE2 IgM-positive patients had higher average body temperature beginning early after hospital admission, followed by elevated CRP and D-dimer measurements. The IgM anti-ACE2–positive group had statistically significantly higher average temperatures, CRP, and D-dimer levels over the first 10 days of hospitalization than the IgM-negative group (*P* = 0.0001, 0.02, and 0.001, respectively). Average absolute neutrophil levels (**G**) were not statistically different between the 2 groups. Analyses in panels **D**–**G** use linear mixed effects model Wald test with 4 degrees of freedom (see *Statistics*). (**H**) Longitudinal analysis of anti-ACE2 IgM antibodies. For all those anti-ACE2 IgM–positive patients with multiple banked sera available (16/18), anti-ACE2 IgM and IgG antibodies were quantitated over time. Red and blue lines on each plot denote anti-ACE2 IgM and IgG antibodies, respectively. Solid black bars represent steroid treatment periods. Additional examples are shown in Supplemental Figure 4.

Anti-ACE2 IgM–positive and –negative patients with COVID-19 did not differ significantly by age, BMI, or sex (Figure 3, A–C). Interestingly, the anti-ACE2 IgM–positive group had statistically significantly higher average temperatures over the first 10 days of hospitalization than the anti-ACE2 IgM–negative group (positive group: mean = 37.5, S^2^ = 0.65, *n* = 783 on M = 20 unique patients, since there were multiple observations on each patient; negative group: mean = 37.0, S^2^ = 0.56, *n* = 3137 on M = 97 unique patients; χ^2^ = 22.72, *P* = 0.0001 from linear mixed effects model Wald test with 4 degrees of freedom) (see *Statistics* and Figure 3D). The results did not qualitatively change when we restricted the analysis to the severe anti-ACE2 IgM–positive patients above and compared them to all severe COVID-19 patients from the CROWN Registry for whom anti-ACE2 IgM status was unknown (IgM-positive: mean = 37.53, S^2^ = 0.64, *n* = 721 on M = 18 unique patients; IgM-unknown: mean = 37.11, S^2^ = 0.59, *n* = 14827 on M = 473 unique patients; χ^2^ = 19.98, *P* = 0.0005 from linear mixed effects model Wald test with 4 degrees of freedom) (see *Statistics* and Supplemental Figure 3). Population average C-reactive protein (CRP) levels were also different between the 2 groups in the first 10 days after admission; this peaked at approximately days 4 to 6 after admission at 20 mg/dL in the anti-ACE2 IgM–positive group, compared with 7.4 mg/dL for the anti-ACE2 IgM–negative group (IgM-positive: mean = 16.96, S^2^ = 104.55, *n* = 95 on M = 18 unique patients; IgM-negative: mean = 13.52, S^2^ = 151.58, *n* = 413 on M = 90 unique patients; χ^2^ = 11.19, *P* = 0.02, from linear mixed effects model Wald test with 4 degrees of freedom) (see *Statistics* and Figure 3E). Similar results were observed for D-dimer levels (Figure 3F), where population average levels peaked at approximately 7.5 days after admission at 6 mg/L in the anti-ACE2–positive group, compared with 2.2 mg/L in the anti-ACE2–negative group (*P* = 0.0014, from linear mixed effects model Wald test with 4 degrees of freedom). While population average neutrophil counts (Figure 3G) were higher in the anti-ACE2–positive group (6.68 K/mm^3^) than in the anti-ACE2–negative group (4.95 K/mm^3^), these results did not reach statistical significance (*P* = 0.10). The elevated temperature observed in anti-ACE2 IgM–positive patients early after admission (Figure 3, D and E) was followed by subsequent enhanced laboratory features of inflammation and coagulation in that disease subgroup, suggesting that the amplification to severe disease in anti-ACE2 IgM–positive patients may begin significantly before decompensation (2).

### Anti-ACE2 IgM does not undergo class-switching to IgG.

Since IgM is the earliest isotype elaborated in immune responses, we pursued a longitudinal analysis of anti-ACE2 IgM on all positive patients for whom serum was available. This demonstrated several patterns: (a) In 3 patients (CV-117, CV-123, CV-128), sampling spanned the development of anti-ACE2 IgM (Figure 3H and Supplemental Figure 4). In these cases, autoantibodies appeared at approximately 10 days after admission and around the time of clinical worsening and intubation. We have not captured sufficient numbers of events around this time to make a definitive statement about onset of antibodies, but they do not appear to significantly precede clinical worsening. (b) Anti-ACE2 IgM autoantibodies were already elevated at the first time point assayed in most patients, when patients were already intubated; in 4 patients (CV-1, CV-58, CV-65, CV-126), levels remained stable over time (one example shown in Figure 3H; additional examples in Supplemental Figure 4). (c) In a third group, anti-ACE2 IgM levels decreased over time (CV-113, CV-124, and CV-134, Figure 3H; CV-3, CV-57, CV-64, CV-129, CV-140, and CV-143, Supplemental Figure 4).

In T cell–dependent immune responses, IgM levels generally decrease at the time of class-switching to IgG. We therefore examined whether decreasing IgM levels over time were associated with increasing anti-ACE2 IgG levels at later points. In 1 patient (CV-1), both IgG and IgM were present at the earliest point and remained constant over time (Figure 3H). In 8 anti-ACE2 IgM–positive patients, we observed a decrease of anti-ACE2 IgM to approximately 50% of original levels over time; all these patients were anti-ACE2 IgG–negative and remained so.

Multiple groups have noted that high levels of anti–SARS-CoV-2 S protein IgG occurring at the time of hospital admission are associated with more severe disease in COVID-19 (e.g., ref. 17). We assayed antibodies against S protein by ELISA and anti-S and –receptor binding domain antibodies by the CoronaChek assay (Supplemental Figure 5). The mean OD values of anti-S antibodies were statistically significantly higher in patients with severe compared with mild COVID-19 (0.68 ± 0.48 vs. 0.23 ± 0.33; mean ± SD, *P* < 0.0001 by unpaired *t* test; Supplemental Figure 5A, left panel). Anti-S IgG levels were also significantly increased in anti-ACE2 IgM–positive COVID-19 patients compared with anti-ACE2 IgM–negative patients (median 0.55 vs. 0.14; *P* = 0.028; Mann-Whitney test; Supplemental Figure 5A, right panel). Using the CoronaChek assay, 8/8 (100%) of anti-ACE2 IgM–positive patients had a positive anti-S IgG result, compared with only 31/58 (53.4%) of anti-ACE2 IgM–negative patients (*P* = 0.017 by Fisher’s exact test; Supplemental Figure 5B). Because anti-ACE2 IgM–positive patients have evidence of a robust antiviral IgG response, failure of the anti-ACE2 IgM to isotype-switch to IgG is not a general feature of anti-ACE2 patients. Instead, these data strongly suggest that the anti-ACE2 IgM immune response is not predominantly driven by T cells (either antiviral or autoreactive), but rather represents a T cell–independent antibody response induced by SARS-CoV-2 infection.

### Properties of IgM autoantibodies recognizing ACE2.

**Figure 4:**
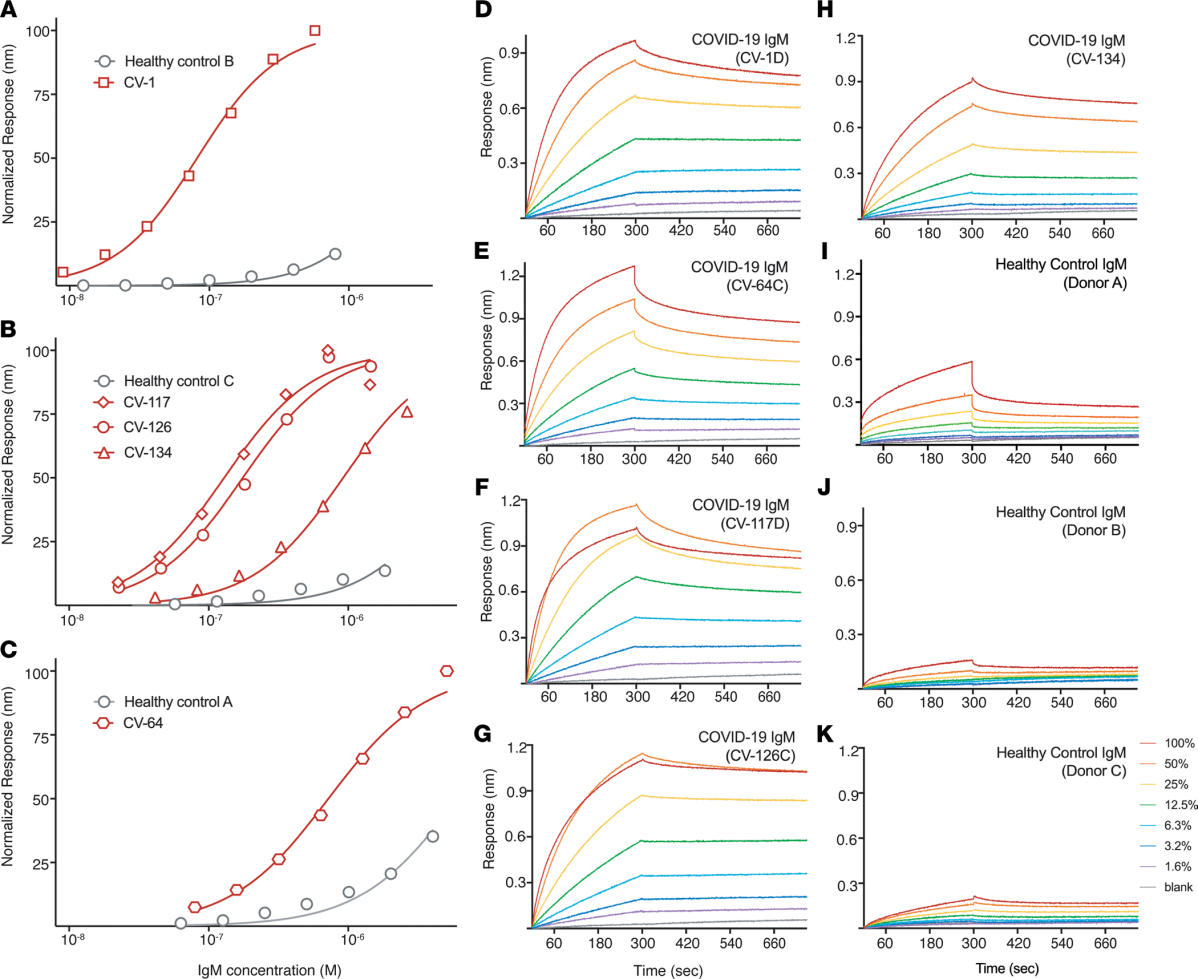
IgM isolated from SARS-CoV-2–infected patients, but not healthy donors, binds to ACE2. Equilibrium binding titrations (**A**–**C**) and kinetic traces (**D**–**K**) of immobilized ACE2 and IgM purified from human serum, as measured by biolayer interferometry. (**A**–**C**) Normalized responses at the indicated concentrations of purified IgM from 3 healthy donors and 5 SARS-CoV-2–infected patients are plotted (data from 3 independent experiments). Equilibrium dissociation constants (*K_D_*) calculated by fitting to a 4-parameter logistic regression model are provided. (**D**–**K**) The indicated percentages (color-coded on the right side of **K**) represent 2-fold dilutions of the 8 purified IgM preparations shown in **A**–**C**. Binding constants were determined via global fitting for each IgM sample using Octet Data Analysis software (assuming a 1:1 binding model). Quantitation of the data shown in **A**–**K** is provided in Supplemental Table 2.

We next pursued analysis of the anti-ACE2 IgM binding properties using a different source of ACE2 antigen and a different assay format. We analyzed IgM purified from 5 patients with high-titer ACE2 antibodies and 3 controls via biolayer interferometry (BLI). Patient IgM binding to immobilized ACE2 was saturable (Figure 4), with apparent *K_D_* values ranging from 5.6 nM (CV-1) to 21.7 nM (CV-134). In contrast, IgM from controls did not approach saturation binding to ACE2 even at the highest tested concentrations (representing the undiluted sample), and the *K_D_* values could thus not be determined (Figure 4 and Supplemental Table 2). It is important to note that the reported *K_D_* values for the anti-ACE2 IgMs provide a lower affinity than the true affinity for the IgM-specific antibodies; since the purified IgM is a mixture of antibodies against various targets, the actual ACE2 affinities for individual IgM clones are presumably higher. Although these IgMs tend to have low monomeric affinity (high nanomolar to micromolar range), they benefit from avidity effects because of their pentameric construction (18).

**Figure 5:**
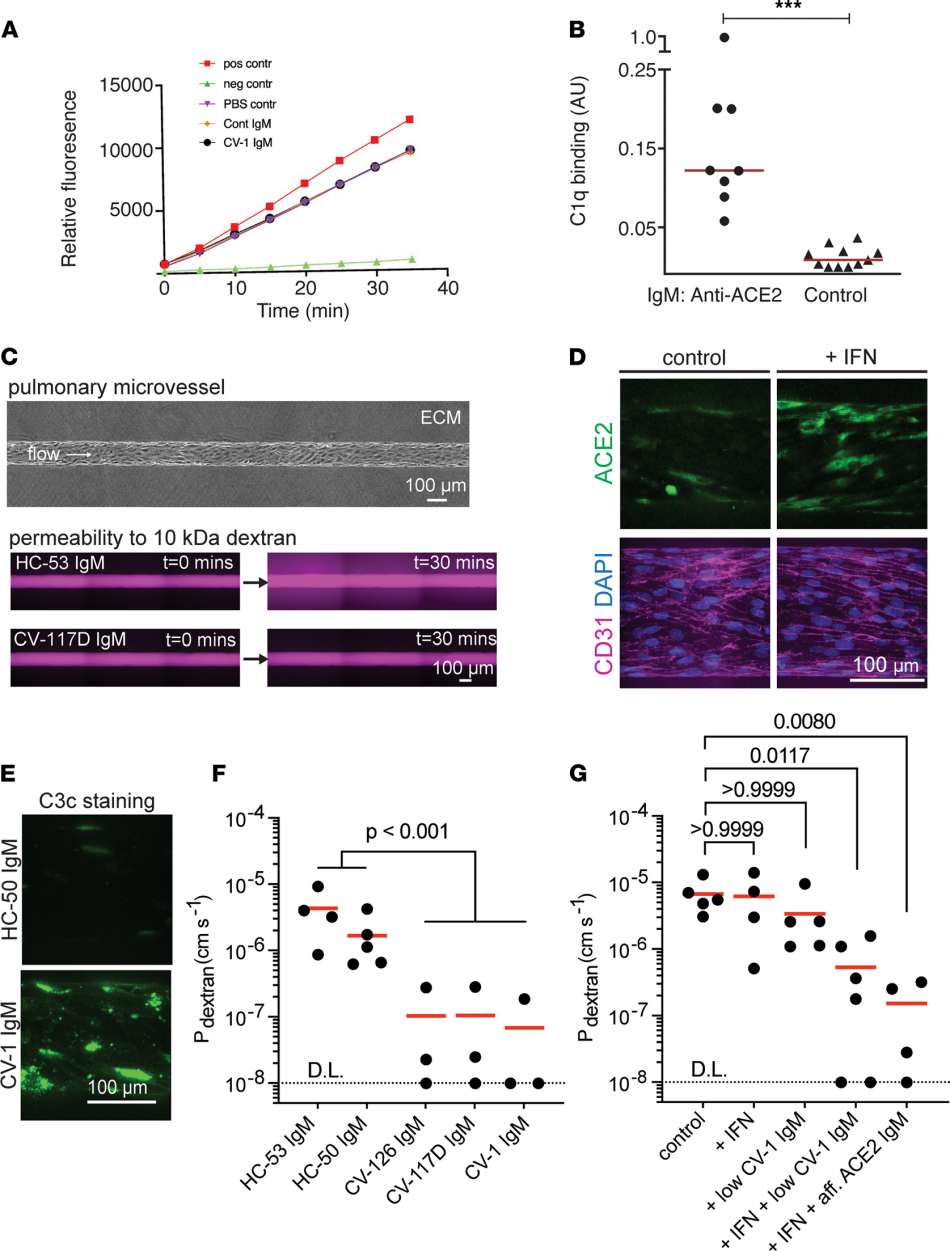
Features of anti-ACE2 IgM antibodies. (**A**) Anti-ACE2 IgM antibodies from patient CV-1 or control do not inhibit ACE2 activity. Positive and negative controls were ACE2 alone, and ACE2 plus ACE2 inhibitor, respectively (see Supplemental Figure 6A). (**B**) IgM antibodies to ACE2 activate complement. Purified IgMs from anti-ACE2 IgM–positive COVID-19 patients (*n* = 8) and healthy controls (*n* = 11) was used for C1q binding assays. Values are means from 2 independent experiments performed on different days. ***P < 0.0001, Mann-Whitney test. (C–G) Anti-ACE2 IgM affects the pulmonary endothelium. (**C**) Phase image of a pulmonary microvessel (top). Fluorescence images of microvessels exposed to anti-ACE2–negative IgM (HC) or anti-ACE2–positive IgM (CV) after perfusion with 10 kDa dextran (lower). Representative images across *n* = 3 to 6 independent experiments for each IgM condition are shown. (**D**) ACE2 and CD31 microvessel staining following 24-hour perfusion with IFN-α/γ. Representative images across *n* = 3 independent experiments are shown. (**E**) C3c staining after perfusion with IFN and anti-ACE2–positive or control IgM (3.33 μg/mL). Representative images across *n* = 3 independent experiments are shown. (**F**) Permeability of microvessels perfused with IFN and anti-ACE2–positive IgM (CV) or anti-ACE2 negative IgM (HC) (100 μg/mL). D.L., detection limit. A linear mixed effects model was used to test the effect of anti-ACE2–positive IgM on permeability. *n* = 3 to 5 for each IgM condition; each dot represents an independent replicate. (**G**) Inhibition of microvessel permeability to 10 kDa dextran in response to anti-ACE2 IgM is IFN dependent. Low CV-1: 3.33 μg/mL; affinity-purified anti-ACE2 IgM: 100 ng/mL. To compare each condition to control, a Kruskal-Wallis test followed by Dunn’s multiple-comparison test was performed. *n* = 4 to 6 for each IgM condition; each dot represents an independent replicate.

Since hypomorphic ACE2 function has been associated with severity in ARDS, we investigated whether purified IgM from patients with COVID-19 affected the catalytic function of ACE2 against a fluorogenic substrate. Two purified IgMs used above in binding assays had no effect on ACE2 activity (Figure 5A, Supplemental Figure 6A, and Supplemental Table 3).

IgM antibodies are mainly found in the circulation, where they are the most effective antibody isotype in activating the classical complement cascade at surfaces expressing their cognate antigens (19). We found that purified IgM antibodies from 8 anti-ACE2 IgM–positive COVID-19 patients consistently bound C1q upon antigen binding (Figure 5B, circles). This was not a feature of IgM purified from 10 anti-ACE2 IgM–negative controls (Figure 5B, triangles). Additionally, C1q binding correlated with levels of anti-ACE2 IgM (Supplemental Figure 6B). These data suggest that IgM antibodies recognizing ACE2 play a role in the complement pathway activation that is prominent in severe COVID-19 patients (20, 21)^.^

### Anti-ACE2 IgM affects the pulmonary endothelium.

Autopsy studies have demonstrated prominent findings in the lungs of COVID-19 patients (22, 23), including enhanced ACE2 expression, endothelial injury, and widespread thrombosis. Interestingly, staining of lung sections for IgM deposition in autopsies from 4 of 17 patients with unknown ACE2 IgM status showed endothelial staining of blood vessels and capillaries (Supplemental Figure 7). We therefore created a tissue-engineered model of a pulmonary microvessel to explore the effects of ACE2 IgM autoantibodies on complement activation and endothelial function. Three-dimensional 150 μm diameter microvessels lined with primary pulmonary microvascular endothelial cells (PMECs) were constructed within type I collagen hydrogel (Figure 5C and Supplemental Figure 8, A and B). Perfusion with IFN-α and IFN-γ for 24 hours increased endothelial membrane expression of ACE2, without altering cell-cell junction morphology (CD31; Figure 5D). Following IFN perfusion, microvessels were treated for 30 minutes under low flow with IgM from individuals who were anti-ACE2 IgM positive or anti-ACE2 IgM negative, both in the presence of 10% human serum as a source of complement (Supplemental Table 3). Perfusion of IFN-treated microvessels with anti-ACE2 IgM, but not control IgM, induced prominent C3c staining, demonstrating that ACE2 IgM induced complement activation in intact microvessels (Figure 5E). Such complement activation may contribute to the endothelial cell injury and microthrombosis observed in patients with severe SARS-CoV-2 infection (20, 21).

Strikingly, exposure to anti-ACE2–positive IgM reduced the permeability of microvessels to 10 kDa dextran by more than 20-fold (*P* < 0.001) (Figure 5F). A decrease in permeability was also observed for Lucifer yellow, a small–molecular weight solute (457 Da) (*P* < 0.01, Supplemental Figure 8, C and D). The permeability of pulmonary microvessels exposed transiently to anti-ACE2–positive IgM returned to baseline levels (similar to permeability of anti-ACE2–negative IgM) by 24 hours after perfusion (Supplemental Figure 8E). Since the residence time (1/k_off_) for CV-1 IgM is about 80 minutes (Supplemental Table 2), we expect most of the anti-ACE2 IgM would be desorbed over 24 hours, confirming the key role of anti-ACE2 IgM binding in regulating the transient increase in barrier function.

To further explore the role of IFN and ACE2 specificity, we compared the effects of IgM dose and affinity-purified anti-ACE2 IgM on barrier permeability (Figure 5G). Perfusion with IFN or anti-ACE2 IgM alone did not induce an increase in barrier function (decrease in permeability). Perfusion with IFN and 3.3 μg/mL of CV-1 IgM induced increases in barrier function (*P* = 0.0117). A similar increase in barrier function was observed after perfusion with 100 ng/mL of affinity-purified anti-ACE2 IgM (*P* = 0.008), providing further evidence that the effect on barrier function is mediated by ACE2 binding. It is noteworthy that some long-lived PMEC stimuli (e.g., sphingosine-1-phosphate exposure) cause an initial tightening of the endothelial barrier, followed by later barrier leakiness mediated through receptor downregulation (24, 25). We hypothesize that prolonged exposure to ACE2 IgM in vivo plays a role in generating the capillary leak that characterizes patients with COVID-19 requiring ventilation.

## Discussion

Overall, these studies demonstrate that anti-ACE2 IgM autoantibodies arise in the context of severe COVID-19, and available evidence suggests that this is likely predominantly a T cell–independent antibody response. The enrichment of these anti-ACE2 IgMs in patients with severe disease, coupled with the ability of anti-ACE2 IgMs to both activate the classical complement cascade and modulate microvascular endothelial barrier properties, strongly suggests that they play a role in the angiocentric, complement-activating pathology observed in patients with severe COVID-19. Anti-ACE2 IgMs demonstrated affinities consistent with those of IgMs that had not undergone somatic hypermutation. ACE2 is clustered in tetraspanin-enriched microdomains with multiple other glycoproteins. We were unable to demonstrate IgM binding to these other colocated antigens, highlighting that the IgM response to ACE2 does not reflect the general targeting of molecules in these structures.

Anti-ACE2 IgM induced striking deposition of C3 and barrier function changes in microvessels lined with PMECs. These effects were only seen when endothelial cells in microvessels were first preincubated with IFN-γ and IFN-α, which are required for inducing surface expression of ACE2. The findings are reminiscent of those made by Pober and colleagues showing that IgM antibodies in sera from patients with acute Kawasaki syndrome caused complement activation and cytotoxicity of IFN-γ–treated human umbilical endothelial cells, but not untreated cells (11).

Importantly, the small amounts of purified anti-ACE2 IgM available only allowed us to address short-term effects of the IgM; when the IgM-containing medium was removed, the enhanced barrier function had fully reversed by 24 hours, consistent with the low affinities of these antibodies. Defining the effects of anti-ACE2 IgM in longer term assays will be essential but must await cloning of ACE2-reactive B cell receptors so that there is sufficient homogeneous material for functional studies.

While our studies did not find an association between anti-ACE2 IgGs and disease severity in COVID-19, papers demonstrate anti-ACE2 IgG are found in COVID-19 and appear to associate with disease severity (26, 27). In both cases, the studies did not distinguish between IgG and IgM, and there were important differences in the assay methodologies used. It is also noteworthy that anti-ACE2 IgGs have previously been observed in established rheumatic diseases (SLE, scleroderma), where these autoantibodies were associated with vasculopathy and pulmonary hypertension (28). The MDA5-associated dermatomyositis phenotype in which we described anti-ACE2 IgM here and before (29) also has a prominent vasculopathy phenotype (16, 30), reminiscent of the features described as COVID toes (31). Future studies to resolve the roles and origins of anti-ACE2 IgM and IgG in COVID-19 severity and vasculopathy will likely provide additional insights into both underlying mechanisms and rational application of novel therapeutics (e.g., calcineurin inhibitors, which appear to decrease severity and mortality in the severe MDA5-associated phenotype; ref. 32).

Anti-ACE2 IgM–positive COVID-19 patients had higher levels of anti-S IgG than those without these antibodies, and patients with these IgMs had higher temperatures early after admission and higher CRP and D-dimer when severe disease became established. In the small number of cases where we could study the appearance of anti-ACE2 IgMs, it was noteworthy that these were first detected around the time of clinical decompensation. Furthermore, when anti-ACE2 IgM levels waned, this was not accompanied by appearance of anti-ACE2 IgGs. This suggests strongly that although these IgMs arise in the setting of a robust antiviral IgG immune response and that the affinity of S protein for ACE2 indicates that complexes of the 2 partners likely exist in vivo (33), the anti-ACE2 IgM response is not driven by anti-S T cell responses. Additional studies to understand the upstream mechanisms that generate these IgM autoantibodies are a high priority.

Circulating IgM autoantibodies manifest an array of effector functions, reflecting their tendency to remain intravascular due to high molecular weight and their capacity to activate the classical complement cascade. This results in chemoattraction and subsequent activation of myelomonocytic cells through various complement split products, as well as terminal complement complex-induced activation and/or damage of cells to which they become bound. Upon formation of the membrane attack complex (MAC), multiple signaling pathways can be initiated (34). These include pathways induced by ion flux across the plasma membrane, including increased intracellular calcium levels that can activate multiple downstream protein kinases, which affect actin cytoskeleton dynamics and organization (34). Additionally, MAC also activates the extracellular signal–regulated kinase (ERK; ref. 35), which influences many downstream targets. Of particular interest is the ERK phosphorylation-induced activation of sphingosine kinases 1 and 2 (36), which generate sphingosine-1-phosphate, a known and potent modulator of junctional permeability through its effects on the actin cytoskeleton (25).

The ability of anti-ACE2 IgMs from patients with COVID-19 to bind C1q and induce C3 deposition on PMECs is consistent with their playing a role in inducing the complement pathway activation that is prominent in patients with severe COVID-19 (20, 21). Directly demonstrating that anti-ACE2 IgMs are associated with plasma biomarkers of endothelial cell activation and damage (including E selectin and von Willebrand factor) and complement activation will be important.

These IgM-activated pathways are potentially amenable to several readily available treatments applied in the acute inflammatory phase of severe illness, e.g., steroids and IVIG therapy (37–39), as well as inhibitors of complement (40) or calcineurin (41). Since anti-ACE2 IgM autoantibodies have features of T cell–independent responses, this may provide an important opportunity to use short-term immune-focused therapies in incipient severe COVID-19 (consistent with the dexamethasone results; ref. 12) rather than the deeper immunosuppression needed for T cell–driven processes. This would be particularly useful in the context of COVID-19 treatment, since T cell responses are needed to preserve effective antiviral activity.

The subset of patients (27%) with severe COVID-19 who developed IgM antibodies against ACE2 (more women, antibodies following infection) are almost certainly not overlapping with the 10% of patients with severe COVID-19 who had preexisting IgG antibodies against type I IFNs (autoantibodies are IgG, almost exclusively in males, preceding infection; ref. 4). These endophenotypes, which have different actionable disease markers, and whose severity is driven by distinct mechanisms, will require distinct therapeutic approaches. In the setting of the ongoing surges of international morbidity and mortality observed with the continuously evolving SARS-CoV-2, defining additional mechanistically anchored groups that are amenable to immune-focused therapies in severe COVID-19 remains a high priority.

## Methods

### Patient data and serum samples.

The study cohort was defined as inpatients who had: (a) a confirmed diagnosis of COVID-19; (b) survival to death or discharge; and (c) remnant specimens in the Johns Hopkins COVID-19 Remnant Specimen Biorepository, an opportunity sample that includes 59% of Johns Hopkins Hospital patients with COVID-19 and 66% of patients with length of stay ≥ 3 days between April 20, 2020, and May 14, 2020. Diagnosis of COVID-19 was defined as detection of SARS-CoV-2 using any PCR test with an Emergency Use Authorization from the US Food and Drug Administration. Selection and frequency of other laboratory testing were determined by treating physicians. The primary clinical data source was JH-CROWN, a Johns Hopkins Medicine COVID-19 registry that integrates all clinical data for COVID-19 patients, including demographics, medical history, comorbid conditions, symptoms, medications, laboratory results, medical images, and comprehensive bedside flow sheet data, including vital signs, respiratory events, and intravenous medication titration (2).

Patient outcomes were defined by the WHO COVID-19 disease severity scale. The WHO scale is an 8-point ordinal scale ranging from ambulatory (1 = asymptomatic, 2 = mild limitation in activity), to hospitalized with mild-moderate disease (3 = room air, 4 = nasal cannula or face mask oxygen), hospitalized with severe disease (5 = high-flow nasal canula or noninvasive positive pressure ventilation, 6 = intubation and mechanical ventilation, 7 = intubation and mechanical ventilation and other signs of organ failure: hemodialysis, vasopressors, extracorporeal membrane oxygenation [ECMO]), and 8 = death. For this study we combined adjacent WHO classes, dividing the inpatient population into 2 groups according to maximum WHO severity: patients who did not require mechanical ventilation (WHO classes 3–5) and those who required mechanical ventilation with or without additional support, such as intravenous pressors, continuous renal replacement therapy, and/or ECMO who survived (WHO classes 6–7) or died (WHO class 8). Serum samples were selected for timing within 24 hours of onset of the maximum WHO class; when multiple samples were available, the specimen closest to the WHO class onset was used. The initial analysis used a random sample of 12 to 20 unique patient specimens from each of the 4 classes meeting the criteria above (depending upon specimen availability for the clinical class). To determine biomarker trajectory, we analyzed an expanded cohort of patients who had 3 to 4 consecutive sera per patient across the course of their hospitalization. Patient selection was determined solely by specimen availability. Where available, additional serum for anti-ACE2 IgM–positive individuals was requested from the remnant biorepository.

### Disease and healthy control sera.

Three autoimmune disease control cohorts consisted of the following: (a) sera from *n* = 25 patients with SLE from the Hopkins Lupus Cohort, (b) sera from *n* = 13 patients diagnosed with systemic sclerosis after evaluation at the Johns Hopkins Scleroderma Center, and (c) *n* = 15 patients with necrotizing myopathy defined by a positive anti-HMGCR antibody status evaluated at the Johns Hopkins Myositis Center. Serum from *n* = 30 patients with influenza diagnosed using the Cepheid Xpert Xpress Flu/RSV assay in the Johns Hopkins emergency departments or inpatient units was studied. Eleven were evaluated in the emergency department and discharged to outpatient care; an additional 19 patients were hospitalized and required oxygen therapy or assisted ventilation (5). A total of 50 plasma samples were selected from patients with pneumonia-associated (primary) ARDS enrolled in the ARDSNet Fluids and Catheters Treatment Trial. Sera from *n* = 30 adult healthy control individuals were also studied.

At the initiation of this study, we were struck by the similar clinical presentation of severe COVID-19 to a dermatopulmonary syndrome, characterized by skin rash, rapidly progressive interstitial lung process with frequent progression to a need for ventilatory support, and a unique vasculopathic phenotype including cutaneous ulcers and digital ischemia (16, 30, 42). This syndrome has been associated with IgG autoantibodies against MDA5 (43). In its fulminant form, this can be viewed as a phenocopy of severe COVID-19, with a high mortality in the absence of treatment with steroids, IVIG, or calcineurin inhibition (32, 44). Serum was available to us from a 42-year-old patient with this MDA5-associated syndrome, who developed symptoms of weakness, rash, fevers, and dyspnea in October of 2011. Her clinical course stabilized with immunosuppression consisting of corticosteroids, tacrolimus, and rituximab over the ensuing 8 years. Strikingly, this index patient had IgM and IgG autoantibodies against ACE2; her serum served as the reference calibrator on all ELISA plates.

### Anti-ACE2 and –SARS-CoV-2 spike ELISAs.

ELISA plate wells were coated overnight with 50 ng of purified protein (recombinant human ACE2; Abcam ab151852; SARS-CoV-2 spike protein S1 subunit; SinoBiological 40591-V08B1) diluted in PBS. For each serum assayed, duplicate wells were coated with protein, and an adjacent well was incubated overnight with PBS (to determine background specific to each sample). For anti-ACE2 IgM ELISA, wells were washed with PBS plus 0.1% Tween (PBST), then blocked with 3% milk/PBST. Primary antibody incubations were performed by diluting sera 1:200 in 1% milk/PBST (overnight, 4°C). For area under the curve plots (Supplemental Figure 1B), serial serum dilutions ranging from 1:100 to 1:3200 were used. Wells were then washed with PBST, followed by incubation with HRP-labeled anti-human IgM (heavy chain specific; Jackson ImmunoResearch 109-035-043) diluted 1:5000 in 1% milk/PBST (1 hour, room temperature [RT]). Color was developed with SureBlue peroxidase reagent (KPL). Reactions were terminated by adding HCl, and absorbances were read at 450 nM. The same anti-ACE2 IgM–positive reference serum was included on each plate, and all absorbances were calibrated relative to this. Anti-ACE2 IgG ELISA was performed as described above, with the following modifications. The concentration of Tween in PBST was 0.05%. Blocking was performed with 5% BSA/PBST, and sera and secondary antibodies were diluted with 1% BSA/PBST. The secondary antibody was HRP-labeled anti-human IgG (Jackson ImmunoResearch 109-036-088), diluted 1:10,000. The cutoff for assigning anti-ACE2 IgM and IgG antibody positivity was determined by assaying sera from 30 healthy controls. The mean ± 3 SDs of these values (0.340 and 0.187 calibrated OD units for anti-ACE2 IgM and IgG antibodies, respectively) was taken as the cutoff for each. The anti-ACE2 ELISA was validated by blotting purified recombinant human ACE2 and using a second source of recombinant human ACE2 (SinoBiological 10108-H08H).

### Anti-SARS-CoV-2 S IgG ELISA.

Assays were performed as described for anti-ACE2 IgG ELISAs, using sera diluted 1:1200 (1 hour, RT).

### CoronaChek assay.

The CoronaChek serologic lateral flow assay (Hangzhou Biotest Biotech) detects IgM and IgG antibodies against the S protein and receptor binding domain of SARS-CoV-2. Studies on positive and negative control specimens from Maryland demonstrated sensitivity of 95% (95% CI 83%, 99%) in convalescent plasma donors an average of 50 days after symptom onset; sensitivity of 100% (95% CI 89%, 100%) in PCR-confirmed hospitalized individuals 15 days after symptom onset; specificity of 100% (95% CI 94%, 100%) in pre-pandemic patients infected with rhinoviruses and other coronaviruses.

### Immunoblots of transfected lysates to detect antibodies against CD9, CD81, and TMPRSS2.

Complementary DNAs encoding full-length FLAG-tagged CD9 and TMPRSS2 (OriGene RC202000 and RC208677, respectively) and CD81 (GenScript OHu19408) were sequence verified. These DNAs were transiently transfected into HEK293T cells (purchased from ATCC) with ExpiFectamin_293 reagent (Thermo Fisher Scientific A14524). After 48 hours, the cells were lysed in buffer A (1% NP-40, 20 mM Tris pH 7.4, 150 mM NaCl, 1 mM EDTA) and a protease inhibitor cocktail. Robust expression of transiently expressed proteins was confirmed by immunoblotting equal protein amounts of lysates electrophoresed on 11% SDS-polyacrylamide gels. After blocking the membranes, primary antibody incubations were performed (overnight, 4^o^C) with a mouse monoclonal anti-FLAG antibody (MilliporeSigma F1804; 1:2500 dilution) to detect CD9, a rabbit monoclonal anti-TMPRSS2 antibody (Abcam ab92323; 1:2000 dilution), or a mouse monoclonal anti-CD81 antibody (Proteintech 66866; 1:3000 dilution). Blotted proteins were detected using HRP-labeled secondary antibodies (Jackson ImmunoResearch 711-036-152 and 115-036-062, 1:10,000 dilution) and chemiluminescence (Pierce). Images were acquired using a Protein Simple Fluorochem-M digital imager. To screen for antibodies against CD9, CD81, and TMPRSS2 in patient sera, immunoblots were performed using lysates made from untransfected cells and from validated transiently transfected cell lysates (10 μg/lane, 12% SDS-polyacrylamide gels). The sera were used at 1:1000 dilution (overnight, 4°C), followed by a mixture of HRP-labeled anti-human IgG and anti-human IgM (Jackson ImmunoResearch 109-036-088 and 109-035-043; diluted 1:10,000 and 1:5000, respectively), with detection as described above.

### Purification of IgM from patient serum and affinity purification of anti-ACE2 IgM.

We equilibrated 0.5 mL of POROS CaptureSelect IgM Affinity Matrix (Thermo Fisher Scientific) with 10 column volumes (CV) of PBS pH 7.2 in a Poly-Prep chromatography column (Bio-Rad). Patient sera (400 μL) were diluted 1:10 in PBS, filtered via centrifugation at 12,000*g* at 4°C for 2 minutes using 0.45 μm spin filters (MilliporeSigma), and loaded onto the column. The column was washed twice with 5 CV of PBS. Bound IgM was eluted with 5 CV of 0.1 M glycine pH 3, neutralized with 0.5 CV of 1 M Tris-HCl (pH 8), exchanged into PBS, and concentrated to match the original serum volume. The 280 nm absorbance of the purified IgM was measured to calculate the IgM concentration, using the extinction coefficient for pentameric human IgM.

Affinity purification of anti-ACE2 IgM from COVID-19 patient CV-1 was accomplished as follows. We incubated 3.5 μg biotinylated ACE2 (SinoBiological 10108-H08H-B) with 50 μL of streptavidin dynabeads (Invitrogen 65305) in 1 mL of buffer A for 25 minutes. After removing unbound ACE2 and washing, the streptavidin dynabead/biotinylated ACE2 mix was added to 7.5 μg purified whole CV-1 IgM (prepared as described above) in 0.4 mL of buffer A. The mixture was rotated for 95 minutes, then washed, and the bound anti-ACE2 IgM was eluted with glycine and neutralized as described above. The eluted anti-ACE2 IgM was buffer exchanged into Endothelial Cell Growth Medium-2 (EC medium; Lonza) and concentrated. Anti-ACE2 IgM binding activity of the affinity-purified preparation was confirmed by ELISA (a 1:100 dilution of the affinity-purified preparation gave OD readings equivalent to a 1:1600 dilution of the whole CV-1 IgM starting preparation).

### BLI analysis of ACE2/IgM interaction.

BLI was performed using an Octet RED96 instrument (Molecular Devices) to measure the interaction of purified IgM to ACE2. Wells of a black, flat-bottom, polypropylene plate (Corning) were loaded with the following samples: 50 nM biotinylated human ACE2 (Sino Biological, 10108-H08H-B); 2-fold dilutions of purified patient IgM; PBSA (PBS pH 7.2 containing 0.1% BSA); and regeneration buffer (0.1 M glycine, pH 3). All samples and buffers were filtered before use. ACE2 and the IgM samples were diluted in PBSA. ACE2 was loaded onto hydrated streptavidin biosensor tips (Molecular Devices), and baseline measurements were collected in PBSA. Binding kinetics were then measured by submerging the ACE2-coated biosensors in wells containing 2-fold serial dilutions of each patient IgM sample for 300 seconds (association) followed by submerging the biosensor in wells containing only PBSA for 450 seconds (dissociation). Analysis and kinetic curve fitting (assuming a 1:1 binding model) were conducted using Octet Data Analysis HT software version 7.1 (Molecular Devices). Normalized equilibrium binding curves were obtained by plotting the response value after the 300 second association phase for each sample dilution and normalizing to the maximum value. Equilibrium binding curves were fitted to a 4-parameter logistic regression model in GraphPad Prism (v9.0) software.

### ACE2 activity assay.

ACE2 activity was measured using a kit from BioVision (K897). Purified IgM (5 μg) or ACE2 inhibitor was preincubated with ACE2 in white 96-well Costar plates (20 minutes, RT), followed by addition of fluorogenic ACE2 substrate per the manufacturer’s protocol. A PBS control was included for each assay. The positive control contained only ACE2 and substrate, and the negative control was ACE2 plus ACE inhibitor and substrate. Fluorescence was measured every 5 minutes after substrate addition in a BMG Labtech FLUOstar Omega plate reader (excitation 355 nm; emission 460 nm). Fluorescence values for wells containing no ACE2 were subtracted from the readings.

### Complement activation assay.

Polystyrene plates (Nunc MaxiSorp, Thermo Fisher Scientific) were coated overnight with 50 ng/well of ACE2 protein (Abcam ab151852) in PBS or with PBS alone. After washing with PBST, the plates were blocked with 1% gelatin/PBST. Purified IgM (0.5 μg/well) diluted in 1% gelatin/PBST was then added to the wells (overnight, 4°C). After washing with PBST, plates with attached IgM were used for 2 different assays. For C1q binding, human serum (1:50 dilution in gelatin veronal buffer) was added as the complement source. After 1 hour at 37°C, wells were washed with PBST, and goat anti-human C1q (Comptech A200) diluted 1:20,000 in PBST was added (1 hour, RT). HRP-labeled anti-goat IgG (Thermo Fisher Scientific A16005) diluted 1:5000 in PBST was used as secondary antibody. To measure anti-ACE2 IgM, HRP-labeled anti-human IgM (heavy chain specific; Jackson ImmunoResearch 109-035-043) diluted 1:5000 in PBST (1 hour, RT) was used. Following addition of SureBlue peroxidase reagent (KPL), reactions were stopped with HCl and absorbances (450 nM) were read.

### Fabrication of pulmonary microvessels.

PMECs (Lonza) were used to generate 3D pulmonary microvessels. PMECs were cultured in EC medium from passages 6 to 8 using routine procedures. Three-dimensional, 150 μm diameter microvessels were fabricated based on published protocols (45). Microchannels were patterned in 7 mg/mL type I collagen (Corning) using nitinol wire, then cross-linked with 20 mM genipin (Wako). PMECs were suspended at 1 × 10^6^ cells/mL in EC medium and seeded into microchannels under static conditions for 30 minutes to promote cell adhesion. Microchannels were then perfused at 3 dyne/cm^2^ for 48 hours in EC medium to form confluent pulmonary endothelium. To replicate the inflammatory state, microvessels were perfused with EC medium supplemented with 1000 U/mL recombinant human IFN-α2 (BioLegend) and 50 ng/mL recombinant human IFN-γ (R&D Systems) for 24 hours.

### Pulmonary microvessel barrier function.

Seventy-two hours after initial cell seeding (24 hours after incubation with IFNs), microvessels were treated with 100 μg/mL patient IgM in EC medium supplemented with 10% normal human complement (Quidel) for 30 minutes under low-flow conditions (~0.3 dyne/cm^2^). Microvessels were then washed out in the above medium minus IgM (30 minutes, 3 dyne/cm^2^). After washout, microvessels were perfused with 200 μM Lucifer yellow (Thermo Fisher Scientific) or 2 μM fluorescently labeled 10 kDa dextran (Thermo Fisher Scientific) in EC medium supplemented with 10% normal human complement to assess paracellular permeability. Additional experiments were performed with CV-1 IgM at 3.33 μg/mL. Experiments were also performed using 100 ng/mL affinity-purified anti-ACE2 IgM; this concentration matches the low experimental dose (3.33 μg/mL total IgM) based on a predicted 3% prevalence of anti-ACE2 IgM in total IgM. Epifluorescence and phase contrast images at original magnification 10× were collected on an inverted microscope (Nikon Eclipse Ti-E) maintained at 37°C for 10 minutes before and 30 minutes after dye perfusion, with images collected every 2 minutes. Fluorescence intensity profiles of a region consisting of the microvessel lumen and surrounding ECM were obtained using ImageJ (NIH). Permeability was calculated as *P* = (*d*/4)(1/*ΔI*)(*dI*/*dt*), where *d* is the vessel diameter, *ΔI* is the initial increase in fluorescence intensity upon luminal filling, and (*dI*/*dt*)_0_ is the rate of increase in fluorescence intensity as solute exits into the gel (46). The total image size was 8200 μm × 670 μm, where permeability was calculated for 10 adjacent regions of interest (ROIs) (820 μm × 670 μm), with the minimum across 3 adjacent ROIs reported to avoid contributions of interstitial leakage of dye into the hydrogel from the inlet and outlet.

### Immunocytochemistry on microvessels.

Microvessels were washed with PBS for 5 minutes, fixed with 3.7% paraformaldehyde (MilliporeSigma) for 15 minutes, blocked with 10% goat serum (overnight, 4°C), then incubated with primary antibody (6 hours, 4°C). Primary antibodies used were rabbit anti-CD31 (Thermo Fisher Scientific RB-10333-P1, 1 μg/mL), goat anti-ACE2 (R&D Systems AF-933, 10 μg/mL), and FITC-conjugated anti-C3c complement rabbit polyclonal antibody (DAKO F0201, 1:75 dilution). After washing with 10% goat serum for 2 hours, the microvessels were incubated (30 minutes, RT) with secondary antibodies (both from Life Technologies, diluted 1:200: Alexa Fluor 647 [A31573 for CD31] and Alexa Fluor 488 [A11055 for ACE2]). Nuclei were visualized with DAPI (Thermo Fisher Scientific). Microvessel cross sections were reconstructed from confocal *Z*-stacks obtained on a swept field confocal microscope system (Prairie Technologies) with illumination provided by an MLC 400 monolithic laser combiner (Keysight Technologies). For ACE2 images, background subtractions were performed in ImageJ with the background subtract plugin and rolling ball radius set at 500 pixels.

### Immunohistochemistry on human lung autopsy paraffin sections.

Autopsies of 23 patients infected with SARS-CoV-2, documented by PCR on a pre- or postmortem nasopharyngeal swab, were examined. Autopsies were consented for and performed on the clinical service with complete examination of chest organs and in situ sampling of remaining organs and tissues, with histology performed on all sites. Lung paraffin sections from COVID-19 autopsy patients were either stained with hematoxylin and eosin or processed as follows. After deparaffinization and rehydration, the sections were immersed in antigen retrieval solution (DAKO) (30 minutes, 98°C). For IgM staining, the sections were blocked with goat serum (30 minutes, RT), followed by incubation with HRP-labeled goat anti-human IgM (Jackson ImmunoResearch 109-035-043) diluted 1:500. Visualization was performed with a liquid DAB substrate-chromagen system (DAKO), and the sections were counterstained with hematoxylin.

### Data and materials availability.

Patient data and autoantibody data will be stored in the JH-CROWN registry, which is housed on the Johns Hopkins Precision Medicine Analytics Platform. Requests for data or materials derived from human samples may be made available, subject to any underlying restrictions on such data and samples, and will require material transfer and data use agreements through Johns Hopkins University.

### Statistics.

The clinical measures used in this analysis are from the COVID-19 JH-CROWN Registry that is actively curated by a team of clinicians, informaticists, and statisticians to ensure data quality. For repeated measures outcomes (e.g., temperature, CRP, BMI), data were checked by making spaghetti plots (47) and visually checking the consistency of observations over time within an individual. The other main data source comprises laboratory measurements of immune status (e.g., IgM or IgG antibodies) that are binary indicators of presence/absence or absorbance levels as described in the immunoassay section.

To compare the rates of IgM antibody positivity between 2 subgroups, we estimated the ratio of the odds of positivity for one subgroup versus the other (OR) and 95% CI. Given the small numbers of patients in some comparator groups, we used a Fisher’s exact test of the null hypothesis that the odds were equal (OR = 1). To compare means of continuous variables with roughly Gaussian distributions (determined using a quantile-quantile plot), we estimated the mean difference and its standard error and used an unpaired 2-tailed *t* test of the null hypothesis that the 2 population means are equal. When we detected a large deviation from Gaussian distribution (for S protein IgG), a nonparametric test (Mann-Whitney *U*) was used instead.

To compare the trajectory of clinical outcomes over time between IgM-positive and IgM-negative groups, we used a linear mixed effects model (48). Variables were transformed to the log scale if their marginal distribution was more nearly symmetric after transformation. The fixed effects included an indicator variable for IgM-positive status, a smooth function of time (natural cubic spline with 3 degrees of freedom), and their interaction. We assumed each person had a random intercept and random smooth trend to account for the likely correlation among repeated observations on individuals. Given this specification, we estimated the smooth curve for the IgM-positive and -negative groups as well as their difference with 95% CIs. We tested the null hypothesis that the 2 population time curves are the same (coefficients for main effect of IgM and interaction of IgM with time all equal to 0) using a Wald’s test statistic that was compared to a χ^2^ distribution with 4 degrees of freedom. The analysis was repeated using natural splines with 2 to 4 degrees of freedom to ensure that the findings were not sensitive to these assumptions.

Comparison of the permeability of Lucifer yellow and 10 kDa dextran between experiments with serum samples from healthy participants and COVID-19 patients was performed using a linear mixed effects model. The permeability was regressed on the participant (healthy control or COVID) with a random intercept to account for a possible correlation among repeated observations for each person. The variance of the random intercepts was taken as 0, indicating no correlation among repeated observations. This assumption was confirmed by comparing the means of the permeability values for the 2 COVID-19 patients and the 3 healthy controls using a 2-tailed *t* test. These results were statistically significant (dextran: *t* = 10.58, *P* = 0.00183; Lucifer yellow: *t* = 4.029, *P* = 0.027). In this case, the mixed model reduced to ordinary linear regression, and the group effect was highly statistically significant (dextran: *t* = 6.75, *P* < 0.001; Lucifer yellow: *t* = 3.57; *P* < 0.01) (Figure 5F).

### Study approval.

These studies on patients with COVID-19 were approved by the Johns Hopkins University Institutional Review Board (JHU IRB) (IRB00251725, IRB00256018, IRB00256547), with a waiver of consent because all specimens and clinical data were deidentified by the Core for Clinical Research Data Acquisition of the Johns Hopkins Institute for Clinical and Translational Research; the study team had no access to identifiable patient data. Patient numbers per analysis are denoted in the figure legends. Informed consent for studies on various control populations was obtained following protocols approved by the JHU IRB (NA_00039294, NA_00039566, NA00007454, NA_00034839, IRB00066509, IRB00091667).

## Author contributions

AR, LCR, SLZ, DRT, FA, JBS, PCS, MD, PMH, ALC, SCR, RER, AP, BTG, CEM, and OL designed research studies. LCR, FA, MITZ, JBS, EKL, NES, JB, SY, RW, AMV, AG, RML, ZG, PCS, JEH, CEM, CAM, OL, LG, LGA, QY, and DH conducted experiments. AR, LCR, DRT, FA, MITZ, JBS, EKL, NES, JB, SY, PCS, SD, LG, LGA, MD, PMH, ALC, SCR, RER, LMS, KZJF, JEH, BTG, CEM, LCS, CAM, and OL acquired data. AR, SLZ, NES, JB, SY, RW, AMV, AG, RML, ZG, PCS, SD, LG, LGA, MD, PMH, ALC, SCR, BTG, and ZW analyzed data. DRT, WAC, ALC, RER, AP, LMS, KZJF, BTG, LCS, and CAM provided data/reagents. AR, LCR, SLZ, DRT, FA, MITZ, JBS, EKL, NES, JB, SY, RW, AMV, AG, RML, ZG, PCS, SD, LG, MD, PMH, WAC, ALC, SCR, RER, AP, LMS, KZJF, JEH, BTG, CEM, LCS, CAM, OL, LGA, QY, and DH wrote/edited the manuscript. SLZ, DRT, and ZW curated data.

## Supplementary Material

Supplemental data

ICMJE disclosure forms

## Version 1 03/29/2022

In-Press Preview

## Version 2 05/09/2022

Electronic publication
