# Supplementary material for: IgM anti-ACE2 autoantibodies in severe COVID-19 activate complement and perturb vascular endothelial function
Source: JCI Insight. 2022 May 9;7(9):e158362. doi: 10.1172/jci.insight.158362 (PMC9090251; doi:10.1172/jci.insight.158362)
Supplement: Supplemental data [file jciinsight-7-158362-s053.pdf]

# SUPPLEMENTAL FIGURE 1

A Anti-ACE2 IgM antibodies in COVID cohorts

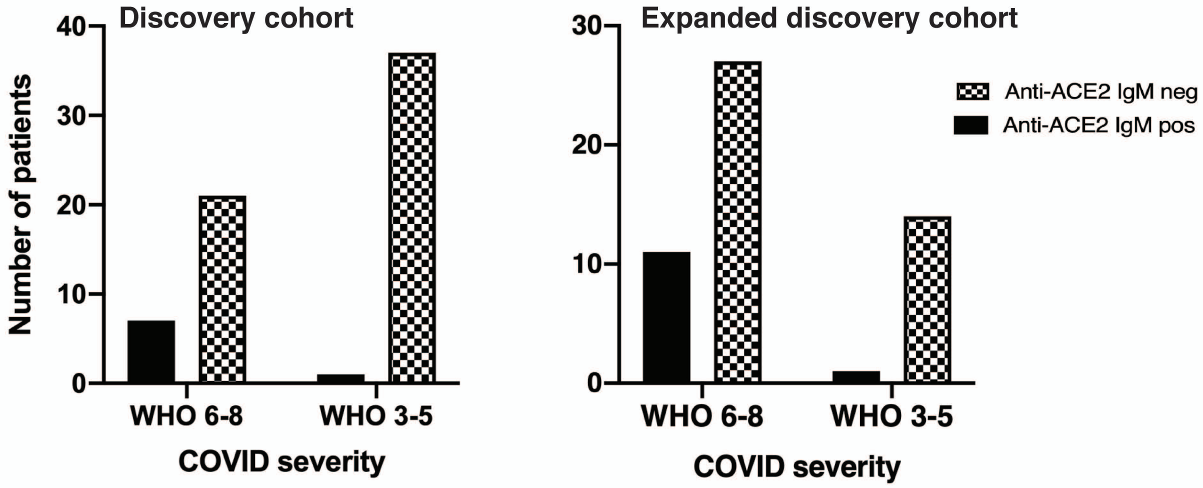


B Anti-ACE2 IgM antibody titrations

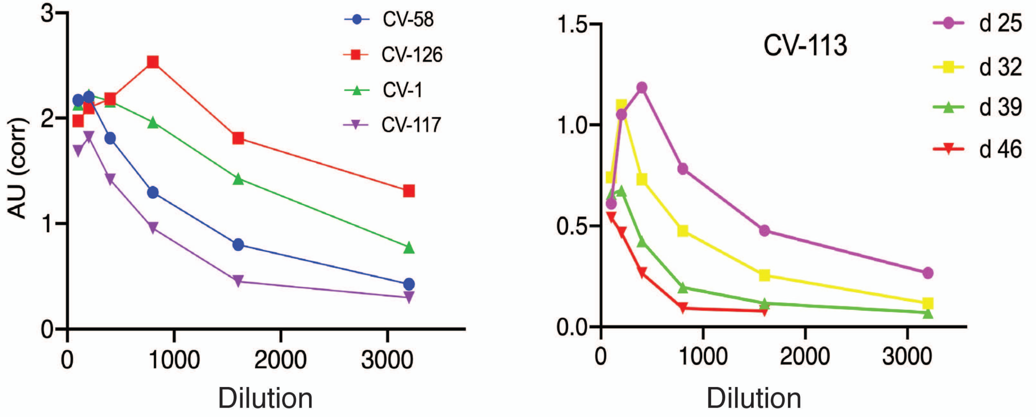

**Supplemental Figure 1: Anti-ACE2 IgM antibodies in COVID-19 patients.** (A): Anti-ACE2 IgM ELISAs were performed as described in the Methods section. In the Discovery cohort (left panel), 8/66 patients with COVID-19 were positive for anti-ACE2 IgM antibodies. Of these, 25% of the WHO 6-8 group were positive compared to 2.6% of the WHO 3-5 group (p=0.0084, Fisher's exact test). An additional 52 COVID-19 patients were assayed ("Expanded discovery cohort", right panel); the frequency of anti-ACE2 IgM in these patients was similar to the initial group (see Fig 2A for data from the combined cohorts; N = 118). (B): Anti-ACE2 IgM ELISAs were performed using serial serum dilutions (1:100 to 1:3,200 range). Data obtained from four different patients is shown in the left panel, each assayed using serum from a single bleed. Data from a fifth patient is shown in the right panel, using serum made from blood draws on 4 different days. Area under the curve ("AUC") plots are shown in both panels. Examples of "raw" optical density readings (obtained at 1:100 and 1:3,200 dilutions) are 2.25 and 0.72 (CV-1 serum) and 1.69 and 0.30 (CV-117 serum).

# SUPPLEMENTAL FIGURE 2

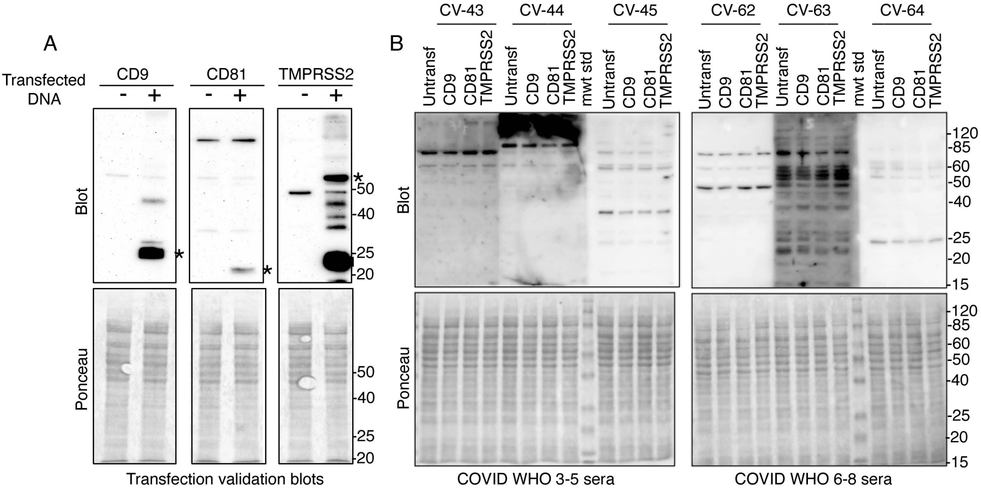

**Supplemental Figure 2: Antibodies against CD9, CD81 and TMPRSS2 are not detected by immunoblotting using sera from patients with COVID-19.** Detergent lysates made from transfected 293T cells were generated as described in the Methods section. (A): Robust expression of transfected CD9, CD81 and TMPRSS2 was confirmed by immunoblotting gel samples from equal protein amounts of untransfected and transfected lysates (10 μg/lane) with relevant antibodies. (B): Serum from COVID-19 patients (diluted 1:1,000) was immunoblotted against panels of untransfected and transfected lysates (10 μg/lane). A mixture of horseradish peroxidase-labeled anti-human IgG and IgM was used for the secondary antibody incubations. Sera from all 66 hospitalized COVID-19 patients in the initial study cohort were assayed; data obtained using sera from 3 patients in WHO ordinal groups 3-5 (CV-43, CV-44 and CV-45), and 3 patients in the 6-8 group (CV-62, CV-63 and CV-64) is shown. (A & B): lower panels show ponceau stained membranes, confirming equal protein loading/lane. Upper panels show immunoblots obtained using the ponceau-stained membranes shown immediately below each. Migration of protein molecular weight marker standards are indicated on the right.

# SUPPLEMENTAL FIGURE 3

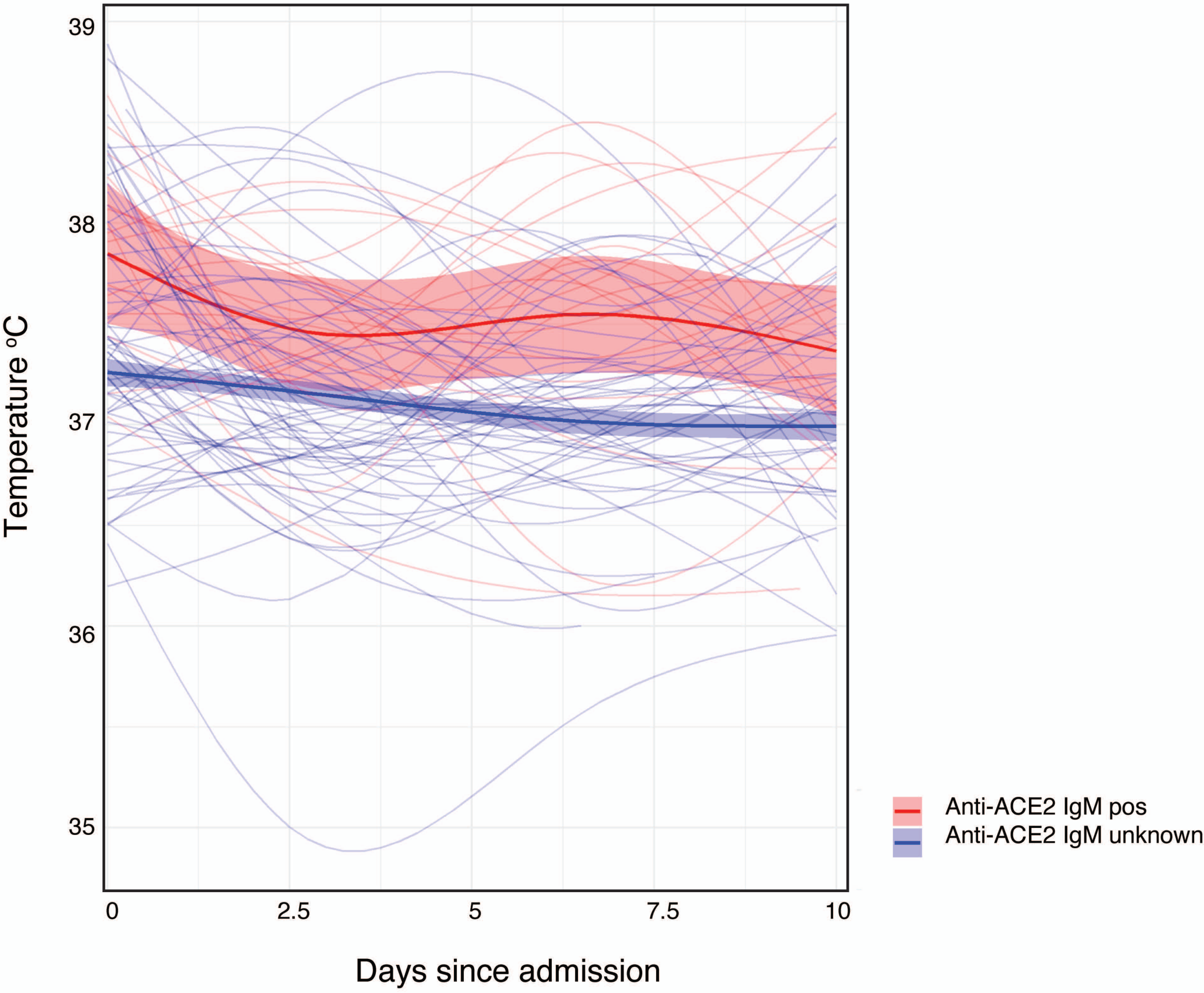

**Supplemental Figure 3: Higher average body temperature measurements in anti-ACE2 IgM-positive patients are not a function of disease severity.** The anti-ACE2 IgM-positive group had statistically significantly higher average temperatures over the first 10 days of hospitalization than the IgM-negative group (see Figure 3D). The analysis here is restricted to the severe IgM-positive patients (denoted in red) compared to all severe COVID-19 patients from the CROWN Registry for whom IgM status was unknown (denoted in blue). The results are unchanged, implicating the increased temperature as a function of IgM status rather than disease severity (IgM-positive: mean = 37.53, $S^2$ = 0.64, N = 721 on M = 18 unique patients, IgM-unknown: mean = 37.11, $S^2$ =0.59, N =14827 on M = 473 unique patients; chisq = 19.98, p = 0.0005 from linear mixed-effects model Wald test with 4 degrees of freedom.

# SUPPLEMENTAL FIGURE 4

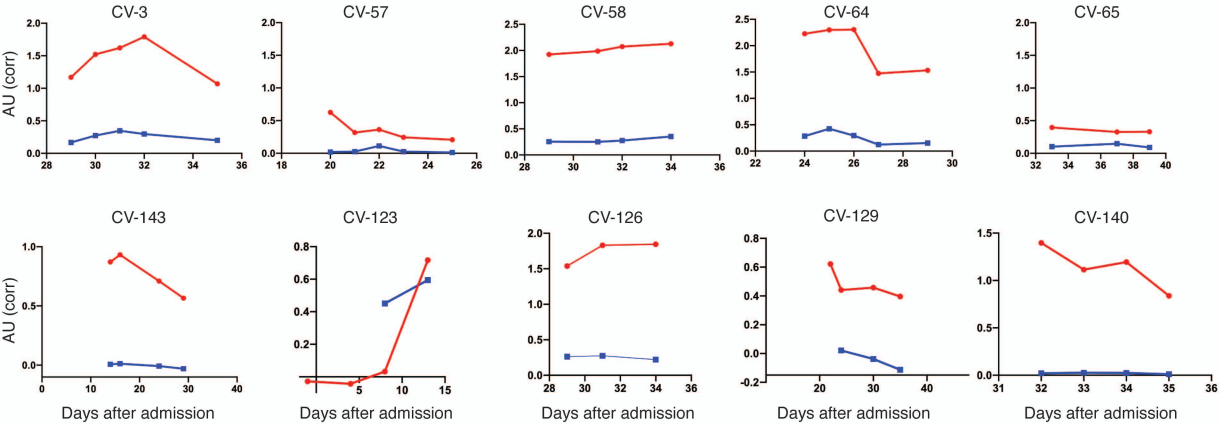

**Supplemental Figure 4: Longitudinal analysis of anti-ACE2 IgM antibodies in patients hospitalized with severe COVID-19**. For all those anti-ACE2 IgM-positive patients with multiple banked sera, anti-ACE2 IgM and IgG antibodies were quantitated over time. Red and blue lines on each plot denote anti-ACE2 IgM and IgG antibodies, respectively. The following patients were on steroid treatment: CV-58 (days 20-24 and 29-36); CV-65 (days 26-28) and CV-129 (day 20 to beyond day 60). Additional examples are shown in Fig. 3F.

# SUPPLEMENTAL FIGURE 5

A SARS-CoV2 IgG spike ELISA

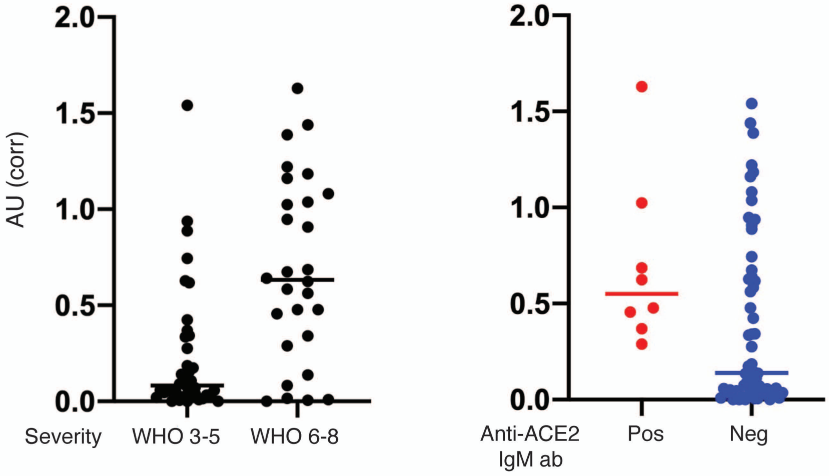


B SARS-CoV2 IgG antibodies - CoronaChek point of care assay

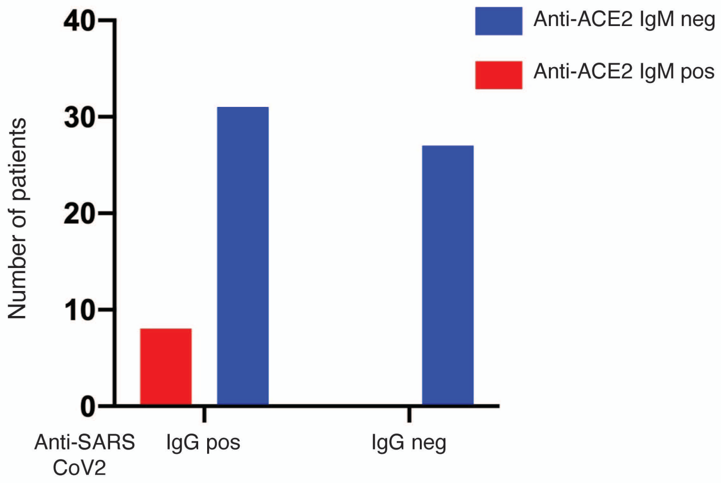

**Supplemental Figure 5: Antibodies against SARS-CoV-2 S-protein in anti-ACE2-positive COVID-19 patients**. (A): Anti-SARS-CoV-2 S-protein IgG antibodies were assayed by ELISA (N=66). Patients are shown grouped by disease severity (left panel), and by anti-ACE2 IgM antibody status (right panel). The mean ODs of anti-Spike (anti-S) antibodies were significantly higher in patients with severe compared to mild COVID ($P<0.0001$, Chi-squared). The median anti-S-antibody level was significantly higher in anti-ACE2 IgM-positive patients compared to anti-ACE2 IgM-negatives ($P=0.028$, Mann-Whitney test). (B): Anti-S and -receptor binding domain (RBD) antibodies assayed by the CoronaChek point of care assay. 8/8 (100%) of anti-ACE2 IgM-positive patients had a positive IgG result, compared to 31/58 (53.4%) of anti-ACE2 IgM-negative patients ($p=0.017$, Fisher's exact test). Red and blue denote anti-ACE2 IgM antibody-positive and -negative patients, respectively.

# SUPPLEMENTAL FIGURE 6

A ACE2 activity assay

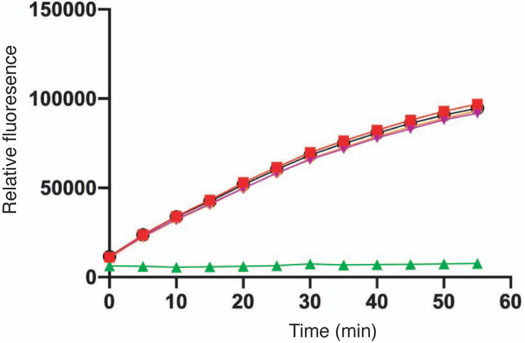


B Correlation of C1q binding and anti-ACE2 IgM

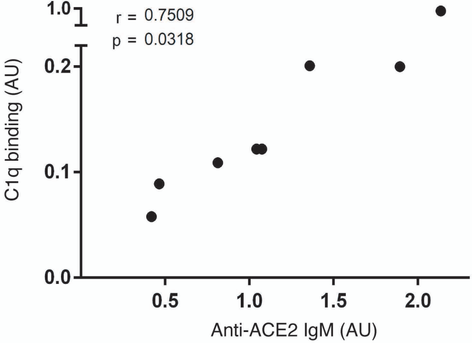

**Supplemental Figure 6: IgM antibodies to ACE2 do not inhibit ACE2 activity (A) and anti-ACE2 levels correlate with C1q binding (B).** (A): ACE2 activity, in the presence or absence of IgM from patient CV-64 or Control A, was measured using a fluorescent substrate in a time course assay. The positive control was ACE2 alone, and the negative control was ACE2 plus ACE2 inhibitor (see Fig 5A for data from CV-1 and control B). (B): C1q binding by anti-ACE2 IgM (see Fig 5B) and levels of anti-ACE2 IgM bound were measured in parallel assays. Values shown are the mean of two independent experiments performed on different days. Pearson correlation $r = 0.7509$, $p = 0.0318$.

# SUPPLEMENTAL FIGURE 7

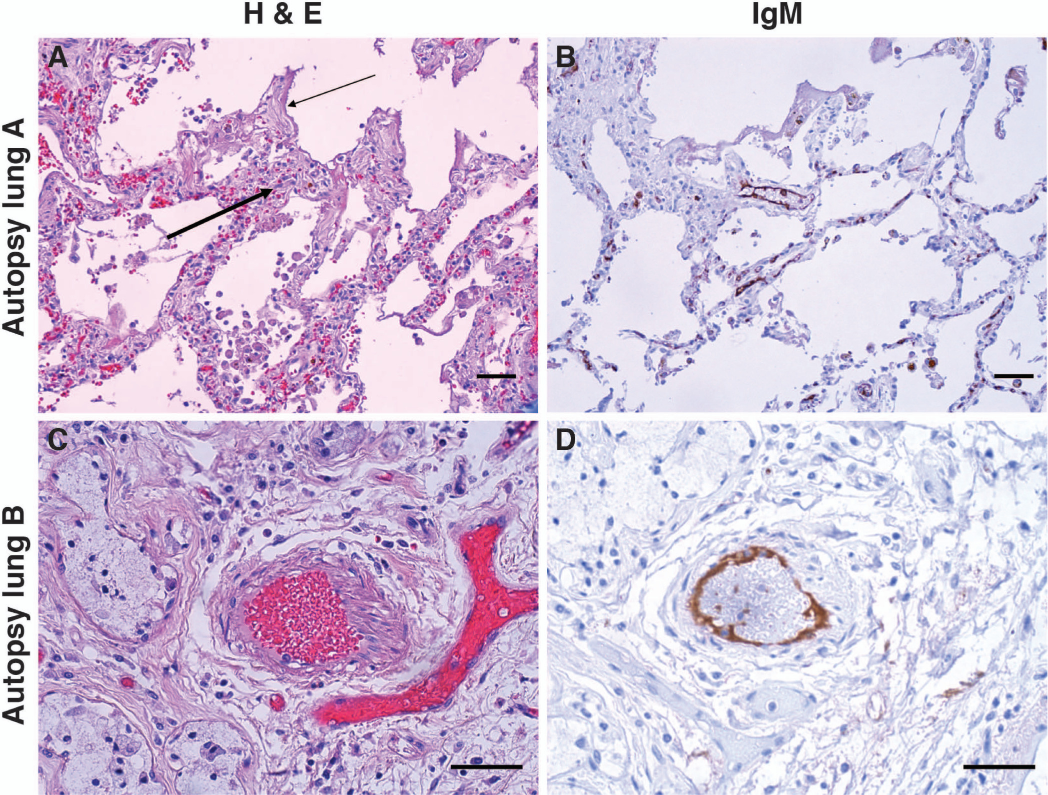

**Supplemental Figure 7: IgM deposition on endothelium in COVID-19 lung.** Lung paraffin sections from two autopsy patients (lung A, upper panels; lung B, lower panels) were stained with hematoxylin and eosin (A & C) or with an anti-IgM antibody (B & D). (A): A section of the left upper lobe of the lung shows a widened interstitium with capillaries showing reactive endothelium (thick arrow). There are hyaline membranes lining alveolar spaces (thin arrow), consistent with the exudative phase of diffuse alveolar damage (acute lung injury). (B): Anti-IgM immunohistochemical staining of the same tissue highlights capillary endothelium in that area. (C): A small artery of a bronchiole stained with hematoxylin and eosin, with (D) endothelial staining for anti-IgM. Size bars represent 50 microns.

# SUPPLEMENTAL FIGURE 8

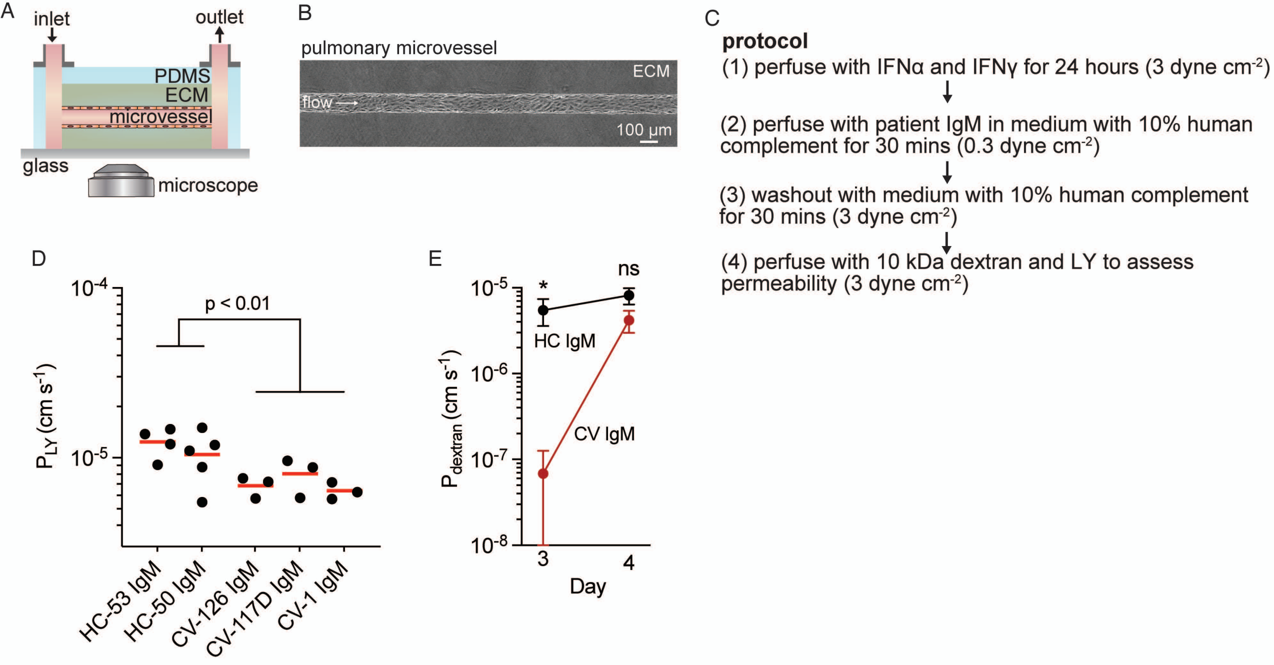

**Supplemental Figure 8: Effects of IgM on tissue-engineered human pulmonary microvessels.** (A): Schematic illustration of microvessel setup. ECM, extracellular matrix; PDMS, polydimethyl siloxane (B): Phase contrast image of a 1 cm-long microvessel at day 3 after seeding pulmonary microvascular endothelial cells into 150 mm-diameter microchannels patterned in type 1 collagen. (C): Summary of experimental protocol to mimic in vivo exposure to IFN, anti-ACE2 IgM, and complement. (D): Permeability of Lucifer yellow in microvessels after 24h perfusion with IFN followed by 30 min perfusion with anti-ACE2-positive IgM (CV) or anti-ACE2-negative IgM (HC) (100 µg/mL). HC, healthy control; CV, COVID-19 patient. For each IgM, 3-5 independent microvessel experiments were performed with two IgM samples from healthy controls and three from COVID-19 patients. A linear random intercept model was used to test the effect of anti-ACE2-positive IgM. The variance of the random intercepts was estimated to be 0, indicating no correlation among repeated observations. In this case, the mixed model reduces to ordinary linear regression and the group effect was statistically significant (lucifer: $t = 3.57$; $p < .01$). (E): Effect of washout on permeability of microvessels to 10 kDa dextran following perfusion with IFN and anti-ACE2-positive IgM (CV) or anti-ACE2 negative IgM (HC) measured on both days 3 and 4 following seeding. Data aggregated across anti-ACE2-positive IgM and anti-ACE2-negative IgM samples (n=3 microvessels), with significance calculated using an unpaired t-test. The permeability of microvessels exposed to anti-ACE2-positive IgM returned to baseline (similar to permeability of anti-ACE2-negative IgM) 24h after perfusion. Size bar, 100 mm

**Supplemental Table 1. Demographics of the study population**

| | | | N |
|---|---|---|---|
| Total | | | 118 |
| | | | |
| **Demographics** | | | |
| Age (years) | 60 (50-71) | | |
| Male gender | | 55% | 66 |
| White race/ethnicity | | 26% | 31 |
| Black race/ethnicity | | 41% | 48 |
| Hispanic race/ethnicity | | 23% | 27 |
| Asian race/ethnicity | | 3% | 3 |
| Other | | 8% | 9 |
| BMI (kg/m2) | 30.2 (26.0-34.9) | | |
| | | | |
| **Comorbidities** | | | |
| Diabetes mellitus | | 43% | 56 |
| Hypertension | | 64% | 76 |
| Coronary artery disease | | 24% | 28 |
| Congestive heart failure | | 23% | 27 |
| Chronic lung disease | | 26% | 31 |
| | | | |
| **Maximum WHO class** | | | |
| Minimal oxygen | | 28% | 33 |
| HFNC/NIPPV | | 15% | 18 |
| Mechanical ventilation | | 32% | 38 |
| Dead | | 25% | 29 |

Continuous variables are median +/- interquartile range

Categorical variables are percentages

**Supplemental Table 2: Kinetic Measurements.** The data shown in Figure 3 was quantitated and is presented below.

| Sample | $K_D$ (nM) | $k_{on}$ (x$10^3$ 1/Ms) | $k_{off}$ (x$10^{-4}$ 1/s) |
|---|---|---|---|
| Healthy A | ND | ND | ND |
| CV-64 | 6.57 ± 0.04 | 22.3 ± 0.3 | 1.46 ± 0.05 |
| Healthy B | ND | ND | ND |
| CV-1 | 5.62 ± 0.02 | 35.5 ± 0.3 | 1.99 ± 0.04 |
| Healthy C | ND | ND | ND |
| CV-117 | 7.70 ± 0.02 | 28.5 ± 0.2 | 2.20 ± 0.03 |
| CV-126 | 10.79 ± 0.02 | 12.8 ± 0.1 | 1.38 ± 0.02 |
| CV-134 | 21.66 ± 0.02 | 9.4 ± 0.1 | 2.04 ± 0.04 |

**Supplemental Table 3**. Summary Table of patient materials studied in various assays using purified IgM shown in the manuscript.

| COVID IgM | anti-ACE2 IgM ELISA * | C1q binding* | biolayer interferometry | Microvessel perfusion assay | ACE2 activity assay |
|---|---|---|---|---|---|
| CV-1 | 1.893 | 0.2 | Yes | Yes | Yes |
| CV-3 | 0.419 | 0.058 | | | |
| CV-58 | 1.073 | 0.122 | | | |
| CV-64 | 1.042 | 0.122 | Yes | | Yes |
| CV-117 | 1.357 | 0.201 | Yes | Yes | |
| CV-124 | 0.812 | 0.109 | | | |
| CV-126 | 2.136 | 0.966 | Yes | Yes | |
| CV-134 | 0.465 | 0.089 | Yes | | |

*denotes absorbance units for ELISA assay and C1q binding performed as described in the Methods section.
